# Supplementary material for: Emergence of a short peptide based reductase via activation of the model hydride rich cofactor
Source: Nat Commun. 2024 May 28;15:4515. doi: 10.1038/s41467-024-48930-w (PMC11130128; doi:10.1038/s41467-024-48930-w)
Supplement: Supplementary file 1 — Supplementary Information [file 41467_2024_48930_MOESM1_ESM.pdf]

Supplementary Information

Emergence of a Short Peptide Based Reductase via Activation of the Model Hydride Rich Cofactor

Ayan Chatterjee[1], Surashree Goswami[1], Raushan Kumar[1], Janmejay Laha[1] and Dibyendu Das[1]*

[1]Department of Chemical Sciences and Centre for Advanced Functional Materials, Indian Institute of Science Education and Research (IISER) Kolkata, Mohanpur 741246, India. Email: dasd@iiserkol.ac.in

Supplementary Methods

Synthetic procedure of peptides

The CEM Liberty Blue peptide synthesizer was used for synthesizing all of the peptides. Fmoc-Rink Amide MBHA Resin (loading 0.52 mmol/g) was swollen using dimethylformamide (DMF) for 30 minutes and subsequently 20% piperidine in DMF was used for Fmoc deprotection. For each Fmoc–amino acid, DIC was used as an activator and the oxyma solution in DMF for coupling step. Thereafter, acetylation of the synthesized peptides was done at free N-terminus (when required) by using acetic anhydride in DMF. Finally, resin bound peptides was washed with DMF and dichloromethane (DCM) respectively and allowed to air dry. From resin, peptides were cleaved using trifluoroacetic acid (TFA)/triethylsilane (5:0.1 V/V) at room temperature for 2 hours. The solution of peptide-TFA mixture was filtered and using high vacuum, TFA was removed. The peptides were precipitated by dropwise addition to ice-cold diethyl ether. The precipitated product was centrifuged at 8000 rpm for 15 min at 4 °C in Eppendorf centrifuge 5804 R resulting in the white pellet. Further, pellet was washed 3 times with cold diethyl ether. The crude peptides were purified using an Atlantis T3 C18 preparative reverse phase column and a preparative Waters HPLC system, a linear gradient of water with 0.1% TFA and acetonitrile with 0.1% TFA was used. Bruker Mass Spec Q-tof systems confirmed the molecular weight.

BLVFFAL-NH_2 (BET-16) ($C_{48}H_{71}N_{11}O_8$) (m/z) calculated for [$M+H^+$]: 808.5177; found: 808.5179.

Ac-RLVFFAL-NH_2 (ARG-16) ($C_{46}H_{70}N_{11}O_8$) (m/z) calculated for [$M+H^+$]: 906.5560; found: 906.5625.

Ac-ELVFFAL-NH_2 (GLU-16) ($C_{45}H_{66}N_8O_{10}$) (m/z) calculated for [$M+Na^+$]: 902.4799; found: 902.4752.

Ac-KLVFFAL-NH_2 (LYS-16) ($C_{49}H_{73}N_{11}O_8$) (m/z) calculated for [$M+H^+$]: 878.5504; found: 878.5554.

Ac-HLVFFAL-NH_2 (HIS-16) ($C_{47}H_{68}N_{10}O_8$) (m/z) calculated for [$M+H^+$]: 887.5150; found: 888.5185.

Ac-RLVFFAR-NH_2 (ARG-16ARG-22) ($C_{46}H_{72}N_{14}O_8$) (m/z) calculated for [$M+H^+$]: 949.5691; found: 949.5662.

Ac-RFLVFAL-NH_2 (ARG-16PHE-17) ($C_{46}H_{71}N_{11}O_8$) (m/z) calculated for [$M+H^+$]: 906.5521; found: 906.5526

Ac-RFF-NH_2 (ARG-18ARG-20) ($C_{26}H_{35}N_7O_4$) (m/z) calculated for [$M+H^+$]: 510.2784; found: 510.2854.

Synthesis of Negatively Charged gold nanoparticle

Negatively charged gold nanoparticles were synthesized from previously published procedure.[1] Briefly, a freshly prepared 20 mL aqueous solution containing 2.5 x 10^{-4} M $HAuCl_4$, and 2.5 x 10^{-4} M trisodium citrate was stirred followed by the addition of 600 μL of ice cold 0.1 M $NaBH_4$ with constant stirring. The solution gradually turned orange-red, indicating the formation of gold nanoparticles with a localized surface plasmon resonance (SPR) transmission at 507 nm. From transmission electron microscopy (TEM) the average size 7 ± 1 nm was measured.

Synthesis of Hexyl 4-nitrobenzoate (1), Ethyl 4-nitrobenzoate (3), Decyl 4-nitrobenzoate (4)

4-Nitrobenzoic acid (2 g, 0.011 mol), Hexanol (1.8 mL, 0.014 mol) (for **1**) / Ethanol (0.8 mL, 0.014 mol) (for **3**) / Decanol (2.74 mL, 0.014 mol) (for **4**) and N,N′-dicyclohexylcarbodiimide (DCC) (2.84 g, 0.014 mol) were dissolved in dry ethyl acetate in a round bottom flask under nitrogen atmosphere.[2] The mixture was stirred for 20 h. Solids were filtered off and further washed with water and saturated aq. $NaHCO_3$. The aqueous phase was extracted with ethyl acetate. Combined organic layers were washed with brine and dried over Na_2SO_4. The final crude product was obtained by removing ethyl acetate under vacuum, then it was purified by column chromatography to get the products.

^{1}H NMR (for **1**) (500 MHz, $CDCl_3$): δ (ppm) 8.27(d, J=11, 2H), 8.19 (d, J=10.95, 2H), 4.35 (t, J=8.4 Hz, 2H), 1.8-1.74 (m, 2H), 1.47-1.39 (m, 2H), 1.37-1.30 (m, 4H), 0.89 (t, J=8.8 Hz, 3H); ^{13}C NMR (for **1**) (100 MHz, $CDCl_3$): δ (ppm) 164.85, 150.58, 135.99, 130.75, 123.60, 66.19, 31.50, 28.64, 25.53, 22.60, 14.05; MS (for **1**) (m/z): [M-H^+] calculated for $C_{13}H_{17}NO_4$, 250.10; found, 250.13.

^{1}H NMR (for **3**) (500 MHz, $CDCl_3$): δ (ppm) 8.29-8.19 (m, 4H), 4.43 (q, J=8.9 Hz, 2H), 1.42 (t, J=8.95 Hz, 3H); ^{13}C NMR (for **3**) (100 MHz, $CDCl_3$): δ (ppm) 164.80, 150.58, 135.95, 130.75, 123.58, 62.05, 14.31; MS (for **3**) (m/z): [M-H^+] calculated for $C_9H_9NO_4$, 194.04; found, 194.00.

^{1}H NMR (for **4**) (500 MHz, $CDCl_3$): δ (ppm) 8.29-8.19 (m, 4H), 4.36 (t, J=6.7 Hz, 2H), 1.79 (dt, J=7.1 Hz, 2H), 1.46-1.26 (m, 14H), 0.88 (t, J=6.8 Hz, 3H); ^{13}C NMR (for **4**) (100 MHz, $CDCl_3$): δ (ppm) 164.8, 150.57, 135.98, 130.73, 123.58, 66.19, 31.95, 29.59, 29.58, 29.36, 29.31, 28.68, 26.05, 22.74, 14.16; MS (for **4**) (m/z): [M-H^+] calculated for $C_9H_9NO_4$, 306.17; found, 306.90.

Synthesis of 2-hydroxyethyl 4-nitrobenzoate (5) and Ethane-1, 2-diyl bis (4)-nitro benzoate (6)

4-Nitrobenzoic acid (2 g, 0.011 mol), ethylene glycol (0.8 mL, 0.015 mol) and N, N′-dicyclohexylcarbodiimide (DCC) (2.84 g, 0.014 mol) were dissolved in dry ethyl acetate in a round bottom flask under nitrogen atmosphere. The mixture was stirred for 20 h. Solids were filtered off and further washed with water and saturated aq. $NaHCO_3$. The aqueous phase was extracted with ethyl acetate. Combined organic layers were washed with brine and dried over Na_2SO_4. The final crude products (mixture of both **5** and **6**) were obtained by removing ethyl acetate under vacuum, then purification by column chromatography was performed to get the products.

^{1}H NMR (for **5**) (500 MHz, $CDCl_3$): δ (ppm) 8.30-8.21 (m, 4H), 4.52-4.50 (m, 2H), 4.00-3.97 (m,

2H); ^{13}C NMR (for **5**) (100 MHz, $CDCl_3$): δ (ppm) 165.9, 150.73, 135.41, 130.93, 130.92, 123.66, 123.64, 67.46, 61.11; MS (for **5**) (m/z): [M+H$^+$] calculated for $C_9H_9NO_5$, 212.05; found, 212.01.
^{1}H NMR (for **6**) (500 MHz, $CDCl_3$): δ (ppm) 8.33-8.30 (m, 4H), 8.23-8.22 (m, 4H), 4.77 (s, 4H); ^{13}C NMR (for **6**) (100 MHz, $CDCl_3$): δ (ppm) 164.55, 150.80, 135.05, 130.93, 123.73, 63.46; MS (for **6**) (m/z): [M+H$^+$] calculated for $C_{16}H_{12}N_2O_8$, 361.06; found, 361.22.

Chemical Structures of Synthesized Substrates

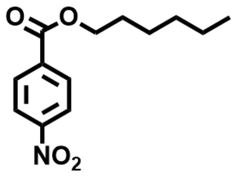

Hexyl 4-nitrobenzoate (1)

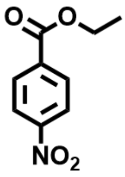

Ethyl 4-nitrobenzoate (3)

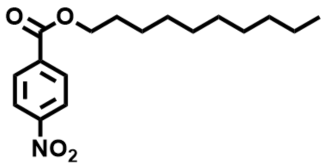

Decyl 4-nitrobenzoate (4)

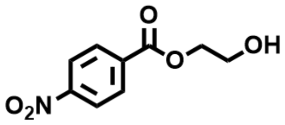

2-hydroxyethyl 4-nitrobenzoate (5)

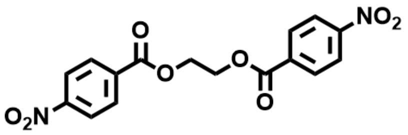

Ethane-1, 2-diyl bis (4)-nitro benzoate (6)

Reaction procedure

Reaction of ester with $NaBH_4$ in the presence of peptide

In a typical experiment, 200 µL of assembled peptides (from 2.5 mM of water redispersed stock solution, final concentration was 500 µM) was added to 790 µL of water, followed by addition of 10 µL of substrate **1** (100 mM stock solution in acetonitrile, final concentration was 1 mM). After 10 min of incubation, 0.38 mg of solid $NaBH_4$ (10 mM concentration in 1 mL of reaction medium) was added to the reaction mixture and was allowed to stir at room temperature for 12 h. Afterwards, the organic parts (unreacted substrate and product) were thoroughly extracted with ether. The ether part was dried by N_2 blowing, followed by the addition of acetonitrile-water for the injection into the high-performance liquid chromatography (HPLC). The product (**2**) of the reaction of **1** by $NaBH_4$ bound BET-16 assemblies after 12 h was collected from HPLC and characterized by mass spectrometry and NMR spectroscopy.

MS ($C_7H_7NO_3$, **2**) (m/z) calculated for [M+H$^+$]: 154.05; found: 154.04.

^{1}H NMR (for **2**) (400 MHz, $CDCl_3$) δ (ppm) 8.22(d, J=11 Hz, 2H), 7.53 (d, J=11 Hz, 2H), 4.83 (d, J=6.2 Hz, 2H) (^{1}H NMR spectra in Supplementary Fig. 7).

To investigate the effect of bound cofactor, at first $NaBH_4$ (10 mM) was incubated with BET-16 (500 µM) assemblies for 30 min followed by centrifugation. The obtained pellet was redispersed in water to

get the preformed complex of cofactor-bound BET-16 assemblies to which the substrate (1 mM) was added. The reaction mixture was allowed to stir for 12 h at room temperature. Further, the organic parts (unreacted substrate and product) were thoroughly extracted with ether. The ether part was dried by N_2 blowing, followed by the addition of acetonitrile-water for the injection into the high-performance liquid chromatography (HPLC).

For recyclability, the aqueous part containing the peptide was centrifuged for 10 min at 12000 rpm to form the pellet. Further, the pellet was washed by water and ether to remove any residual unreacted starting materials. Afterwards, the pellet was redispersed in water and then the above procedure was repeated for next reaction cycle.

pH dependent studies were conducted by using few microlitres of HCl and NaOH for lower and higher pH (the pH for the reaction was found to be 10 due to presence of $NaBH_4$)[3] to maintain desirable pH after addition of all the components of the reactions. The pH was found to remain unaltered throughout the course of the reaction. Notably, pH value below 9.5 could not be achieved due to hydrolysis of $NaBH_4$ in aqueous medium.[3]

Reaction of ester with $NaBH_4$ in presence of CTAB or TBAB

In a typical experiment, 200 µL of CTAB or TBAB (2.5 mM of stock solution in water, final concentration was 500 µM) was added to 790 µL of water, followed by addition of 10 µL of substrate (100 mM ACN stock solution, final concentration was 1 mM). After 10 min of incubation, solid $NaBH_4$ (10 mM concentration in 1 mL of reaction medium) was added to the reaction mixture and was allowed to stir at room temperature for 12 h. Afterwards, the organic parts (unreacted substrate and product) were thoroughly extracted with ether. The ether part was dried by N_2 blowing, followed by the addition of acetonitrile-water for the injection into the high-performance liquid chromatography (HPLC).

Reaction of ester (6) by peptide-$NaBH_4$ or $LiAlH_4$

In a typical experiment, 200 µL of assembled peptides (2.5 mM of water redispersed stock solution, final concentration was 500 µM) was added to 790 µL of water, followed by addition of 10 µL of substrate **6** (100 mM ACN stock solution, final concentration was 1 mM). After 10 min of incubation, 0.38 mg of solid $NaBH_4$ (10 mM concentration in 1 mL of reaction medium) was added to the reaction mixture and was allowed to stir at room temperature for 12 h. Afterwards, the organic parts (unreacted substrate and product) were thoroughly extracted with ether. The ether part was dried by N_2 blowing, followed by the addition of acetonitrile-water for the injection into the high-performance liquid chromatography (HPLC).

For $LiAlH_4$ mediated reaction, 10 µL of **6** (1 M ACN stock solution, final concentration was 1 mM) was added in 9.99 mL of dry THF in a 3-necked flask (total reaction volume 10 mL) in nitrogen environment. Afterwards, total 3.8 mg of solid lithium aluminum hydride (final concentration was 10 mM) was added to the reaction mixture in four parts (0.95 mg), each after an interval of 1 h and then this mixture was allowed to stir for total 12 h. Following this, the flask was put in ice bath for cooling the reaction mixture. Thereafter saturated aqueous sodium bicarbonate was added to quench the excess reagent. Afterwards, the organic parts were extracted with ether (2 x 10 mL) and evaporated by Rota vapor.

Subsequently, 50% (v/v) acetonitrile-water was added for the injection into the HPLC.

Reaction of ester with NADH in the presence of peptide

200 µL of assembled peptides from 2.5 mM of water redispersed peptide stock solution (final concentration was 500 µM) was added to 790 µL of water, followed by addition of 10 µL of substrate **1** (from 100 mM stock solution in acetonitrile, final concentration was 1 mM). After 10 min of incubation, 6.6 mg of solid NADH (10 mM concentration in 1 mL of reaction medium) was added to the reaction mixture and allowed to stir at room temperature for 12 h. Afterwards, the organic parts (unreacted substrate and product) were thoroughly extracted with ether. The ether part was dried by N_2 blowing, followed by the addition of 50% (v/v) acetonitrile-water for the injection into the HPLC.

Supplementary Figures

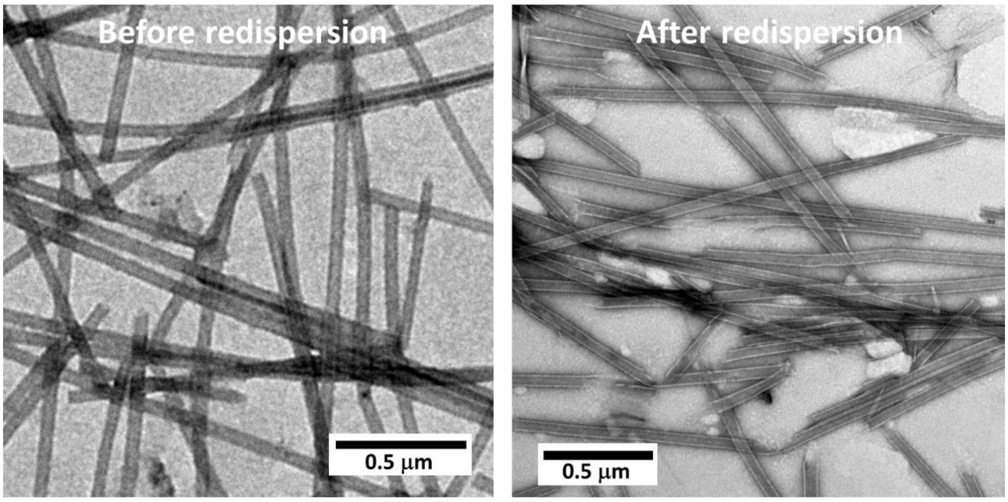

Supplementary Figure 1. **Comparison of ARG-16 nanotubes before and after redispersion.** TEM micrographs of ARG-16 nanotubes before (aged in (v/v) 40% ACN/water containing 0.1% TFA) and after redispersion in water. The experiments were repeated for at least three times.

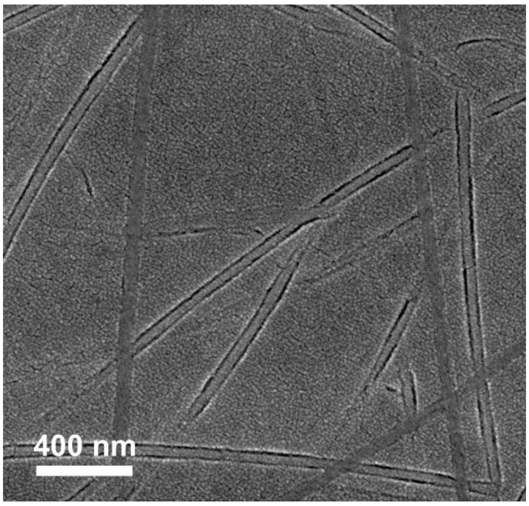

Supplementary Figure 2. **Morphology of ARG-16 nanotubes.** SEM micrograph of ARG-16. The experiment was repeated for at least three times.

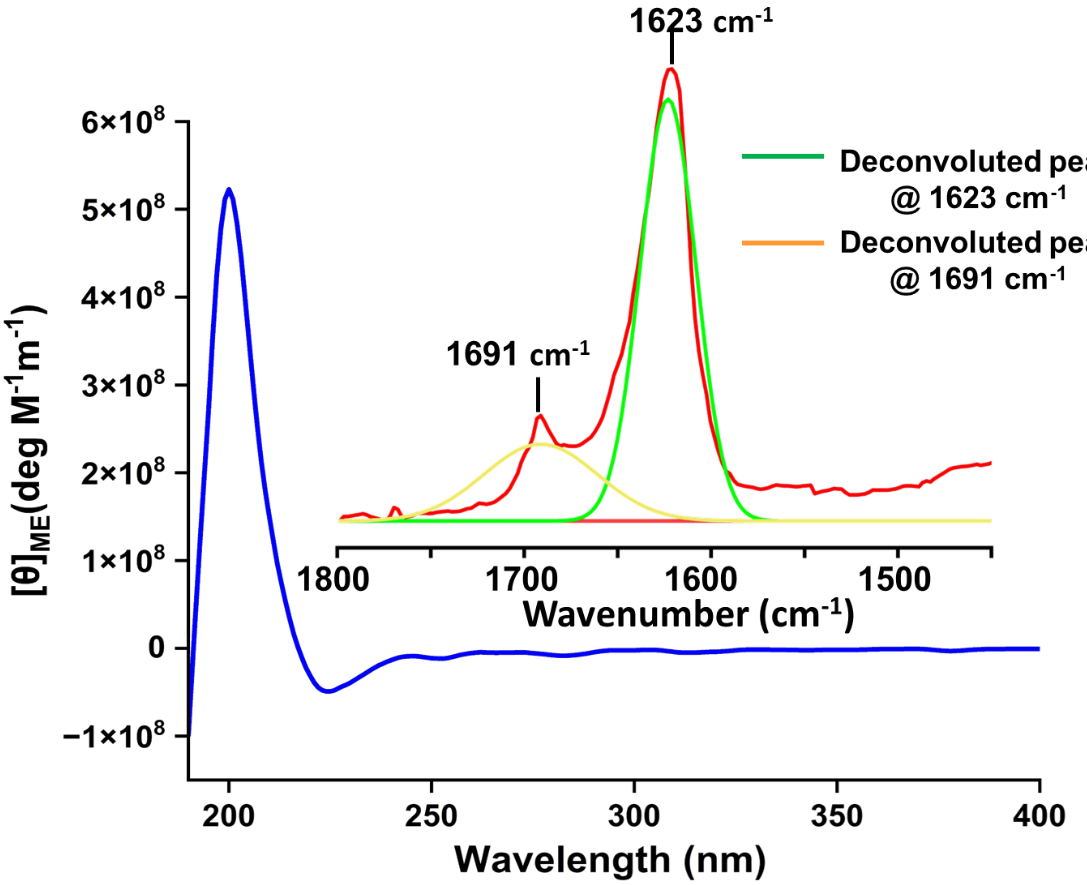

Supplementary Figure 3. **Characterization of ARG-16 assemblies**. CD spectra and FTIR (deconvoluted at amide I region, between 1600 and 1700 cm^{-1}) profile of ARG-16 assemblies. Source data are provided as a Source Data file.

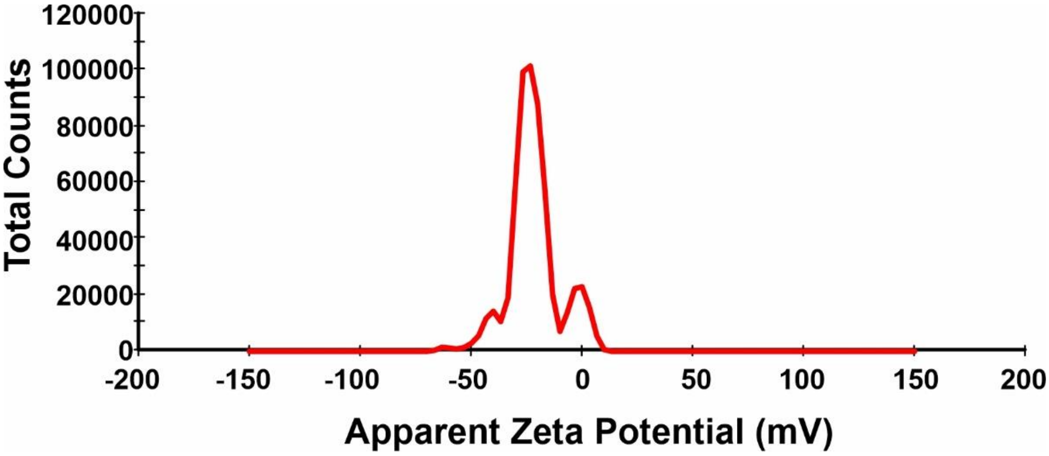

Supplementary Figure 4. **Characterization of the negatively charged gold nanoparticles.** Zeta potential of negatively charged gold nanoparticles.

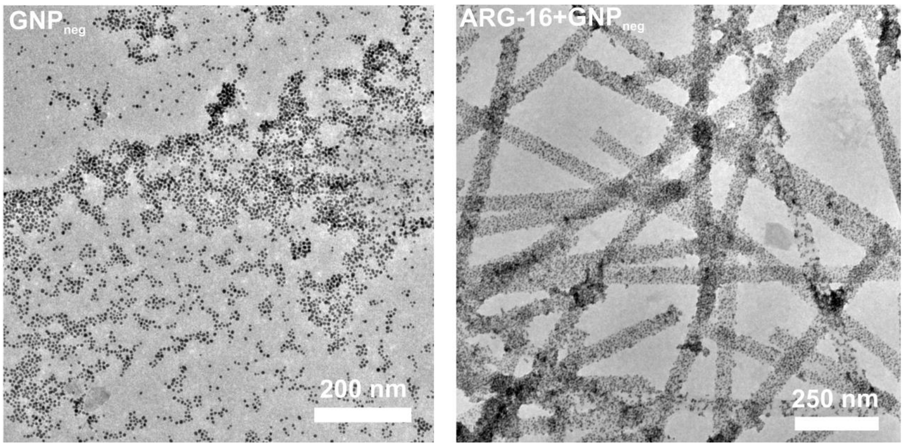

Supplementary Figure 5. Comparison of TEM images of GNP_{neg} in absence or presence of ARG-16 nanotubes. TEM micrographs of negatively charged citrate capped gold nanoparticles in absence (left) and in presence of ARG-16 (right). All the experiments were repeated for at least three times.

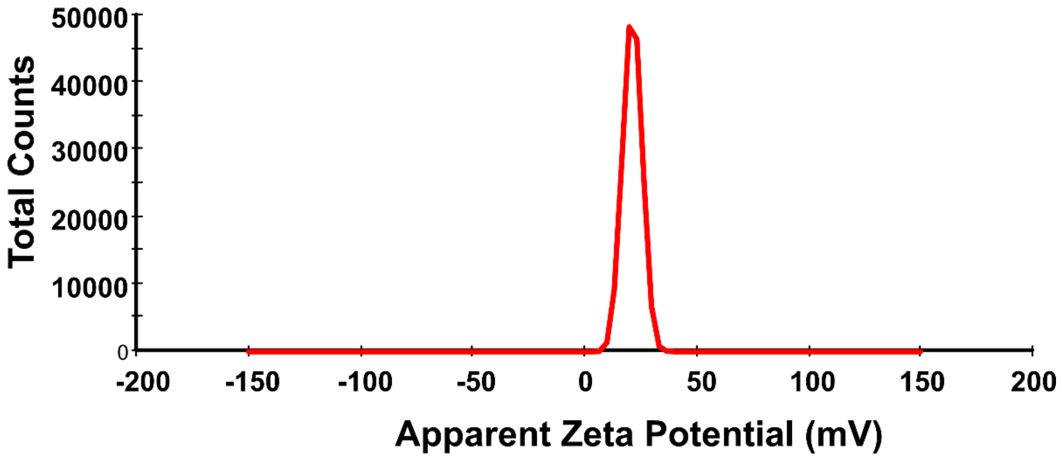

Supplementary Figure 6. **Characterization of ARG-16 nanotubes in presence of GNP_{neg}.** Zeta potential of GNP_{neg} bound ARG-16 assemblies.

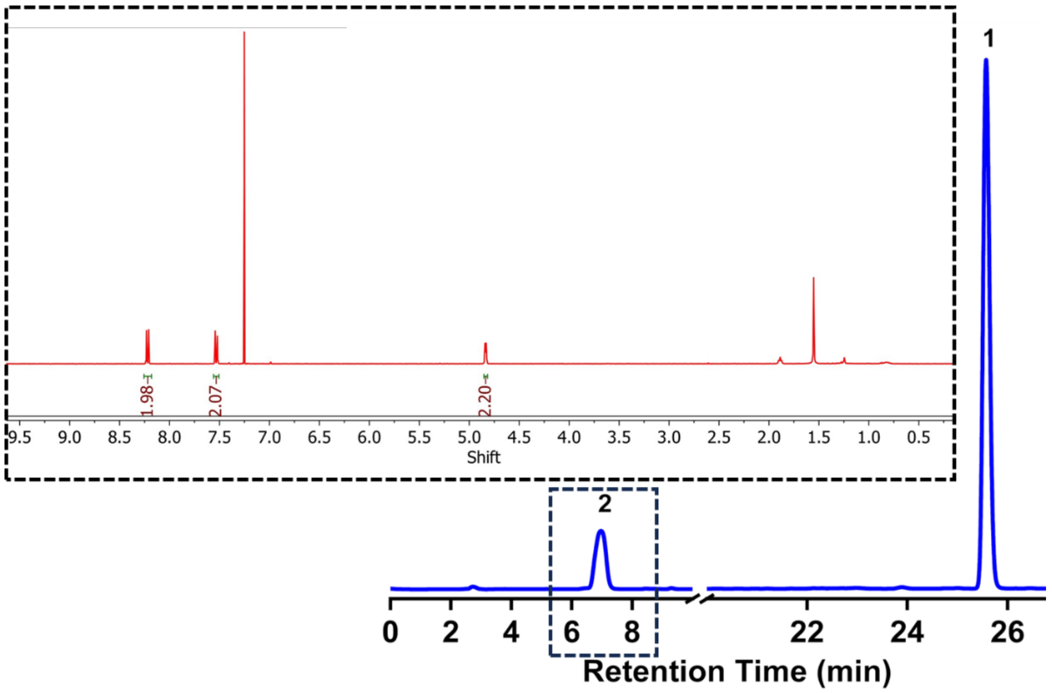

Supplementary Figure 7. Characterization of the product of reduction of 1 by ARG-16-$NaBH_4$ system. HPLC chromatogram of the reduction of **1** by ARG-16-$NaBH_4$ system after 12 h, extracted at 276 nm. Inset: ^{1}H NMR spectra of **2**. Source data are provided as a Source Data file.

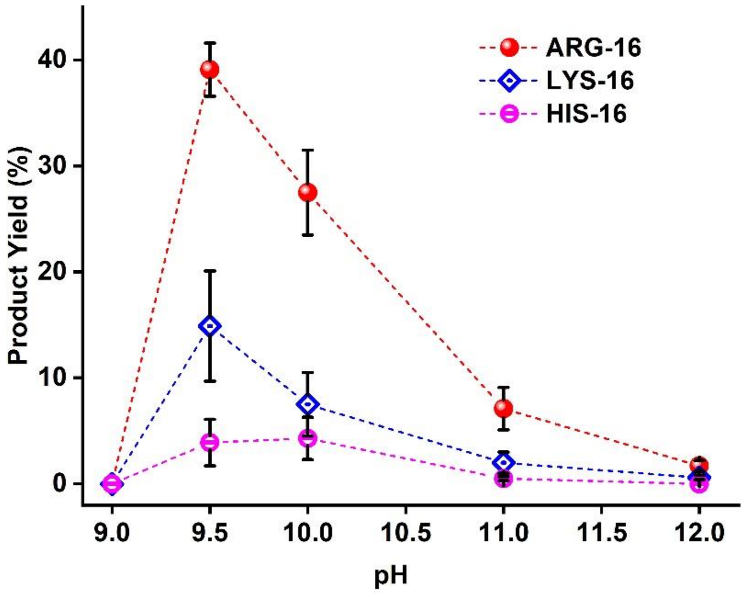

Supplementary Figure 8. **The effect of pH on the yield of 2 by various cofactor bound short peptide-based assemblies.** The variation of the yield of product **2** at different pH by cofactor bound ARG-16, LYS-16 and HIS-16 assemblies. NaOH/HCl was added to adjust the respective pH value of the reaction mixture. The error bars are calculated from three separate experiments. Data are presented as the mean ± s.d. (n=3 independent experiments). Source data are provided as a Source Data file.

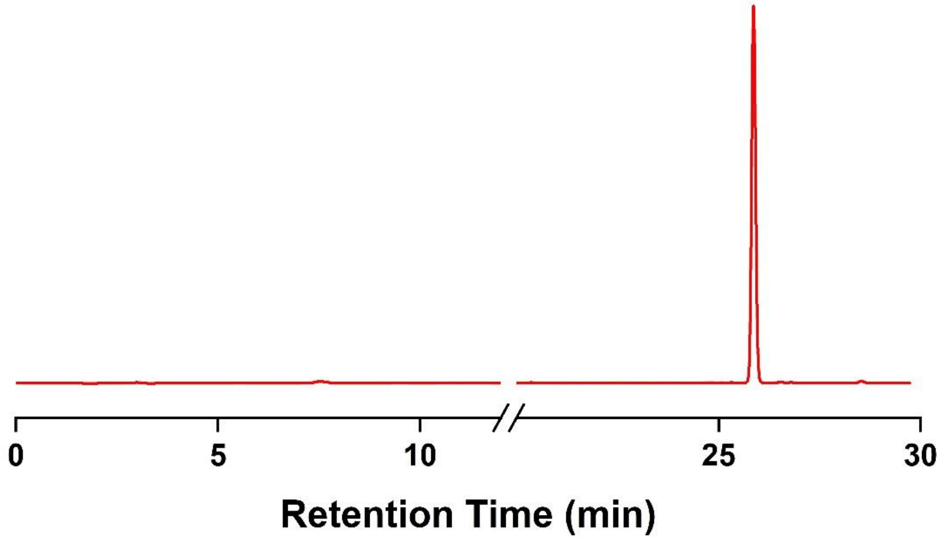

Supplementary Figure 9. **Characterization of the reduction of 1 by ARG-16-$NaBH_4$ system at pH 7 from HPLC analysis.** HPLC chromatogram of the reduction of **1** by ARG-16-$NaBH_4$ system at pH 7 after 12 h, extracted at 276 nm. Source data are provided as a Source Data file.

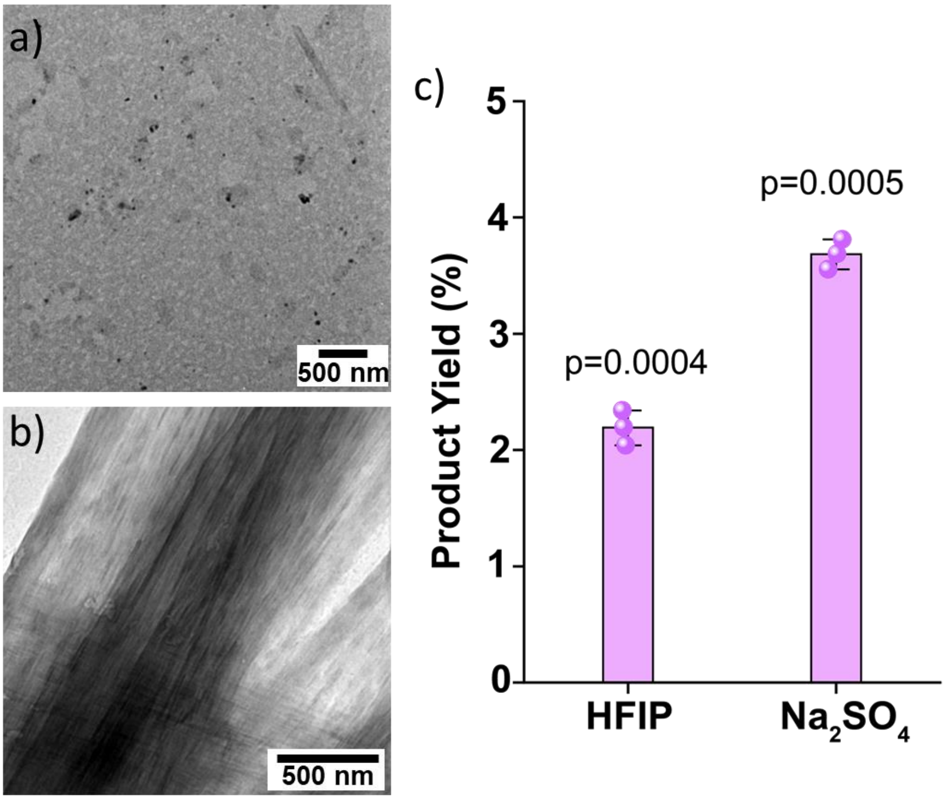

Supplementary Figure 10. **Characterization and the effect of disassembly and bundling of ARG-16 nanotubes on the product yield of reduction of 1.** TEM micrographs of ARG-16 treated with a) HFIP and b) Na_2SO_4. All the experiments were repeated for at least three times. c) Bar diagram showing the yield of product **2** by ARG-16-HFIP-$NaBH_4$ and ARG-16-Na_2SO_4-$NaBH_4$ systems at 12 h. Significance for the two systems was estimated using a two-sided homoscedastic ‘t’ test (*$p \leq 0.05$, **$p \leq 0.01$, ***$p \leq 0.001$) in comparison with the ARG-16-$NaBH_4$ system taken as reference. Data are presented as the mean ± s.d. (n=3 independent experiments). Source data are provided as a Source Data file.

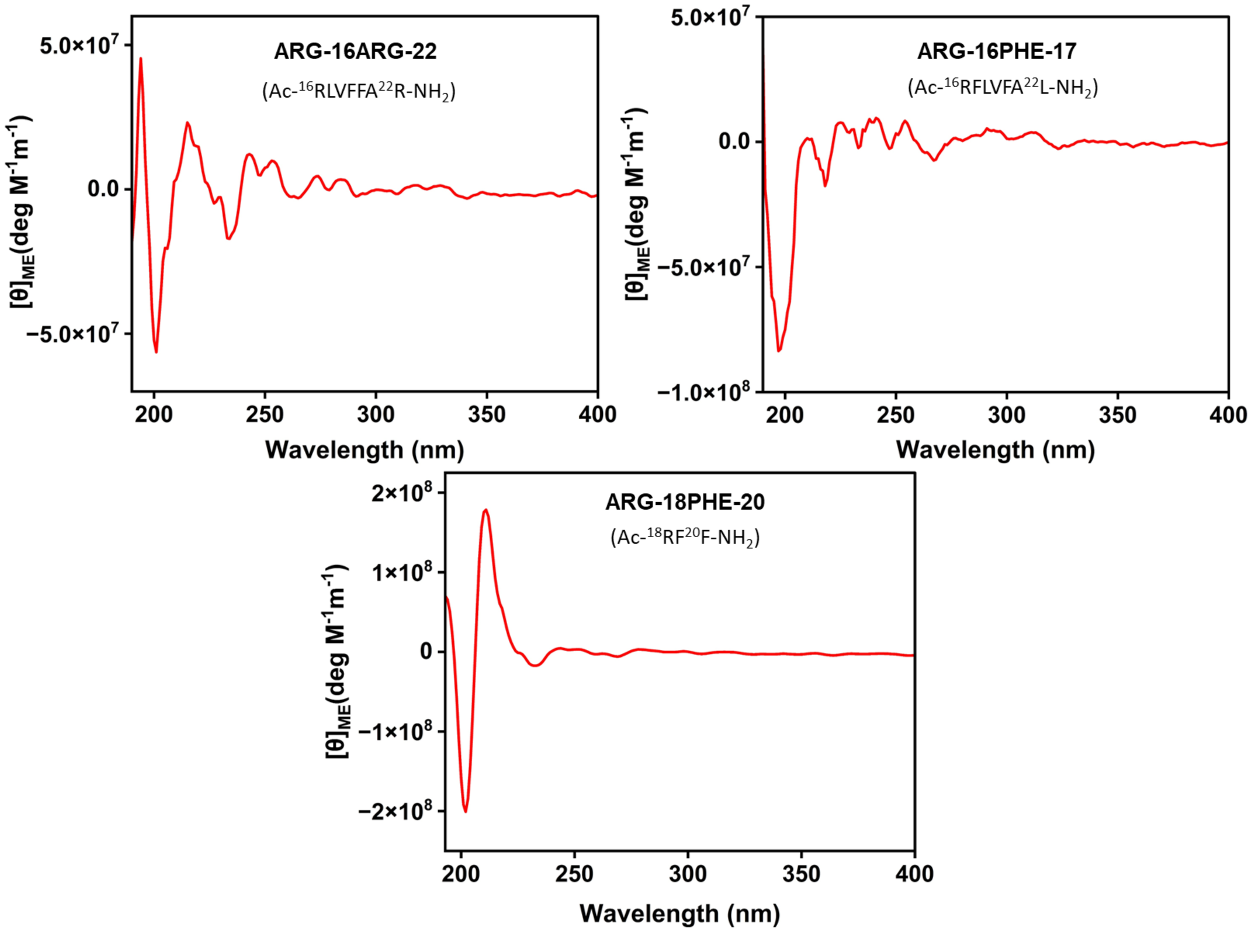

Supplementary Figure 11. **Characterization of different short peptide-based assemblies from CD spectrocsopy.** CD spectra of ARG-16ARG-22, ARG-16PHE-17 and ARG-18PHE-20 assemblies. Source data are provided as a Source Data file.

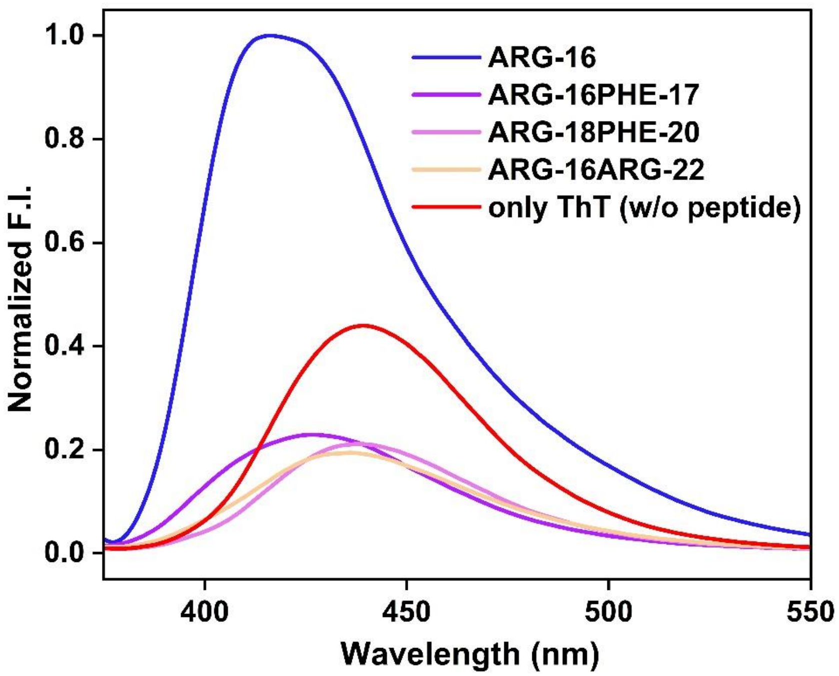

Supplementary Figure 12. **Characterization of different short peptide-based assemblies from ThT assays.** Fluorescence spectra of ThT in absence or presence of ARG-16, ARG-16ARG-22, ARG-16PHE-17 and ARG-18PHE-20 assemblies. Source data are provided as a Source Data file.

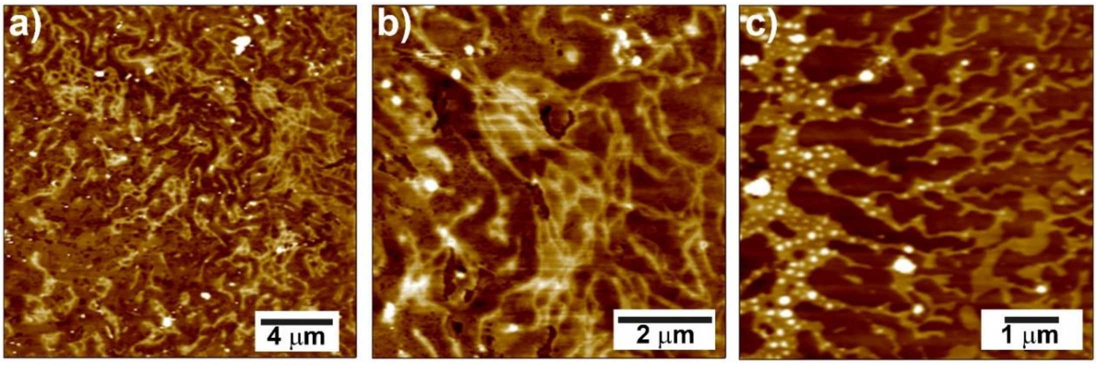

Supplementary Figure 13. **Characterization of different short peptide-based assemblies from AFM microscopy.** AFM micrographs of a) ARG-16ARG-22, b) ARG-16PHE-17, c) ARG-18PHE-20. All the experiments were repeated for at least three times.

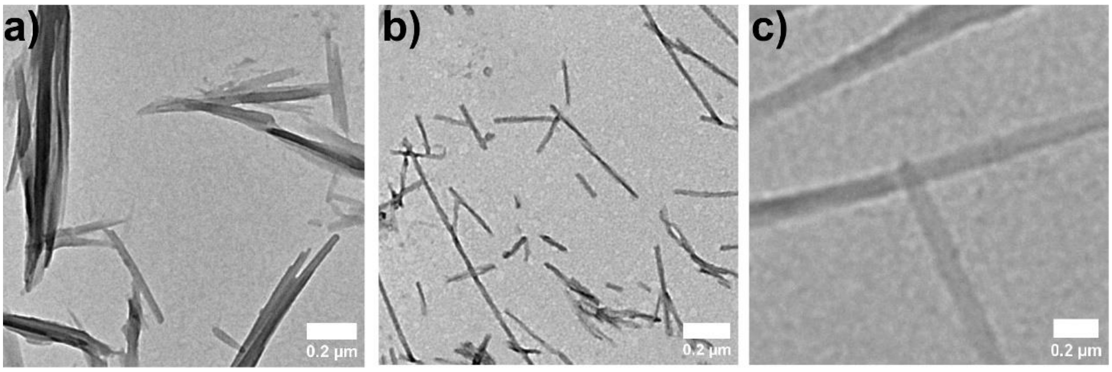

Supplementary Figure 14. Characterization of different short peptide-based assemblies from TEM microscopy. TEM micrographs of a) ARG-16ARG-22, b) ARG-16PHE-17, c) ARG-18PHE-20. All the experiments were repeated for at least three times.

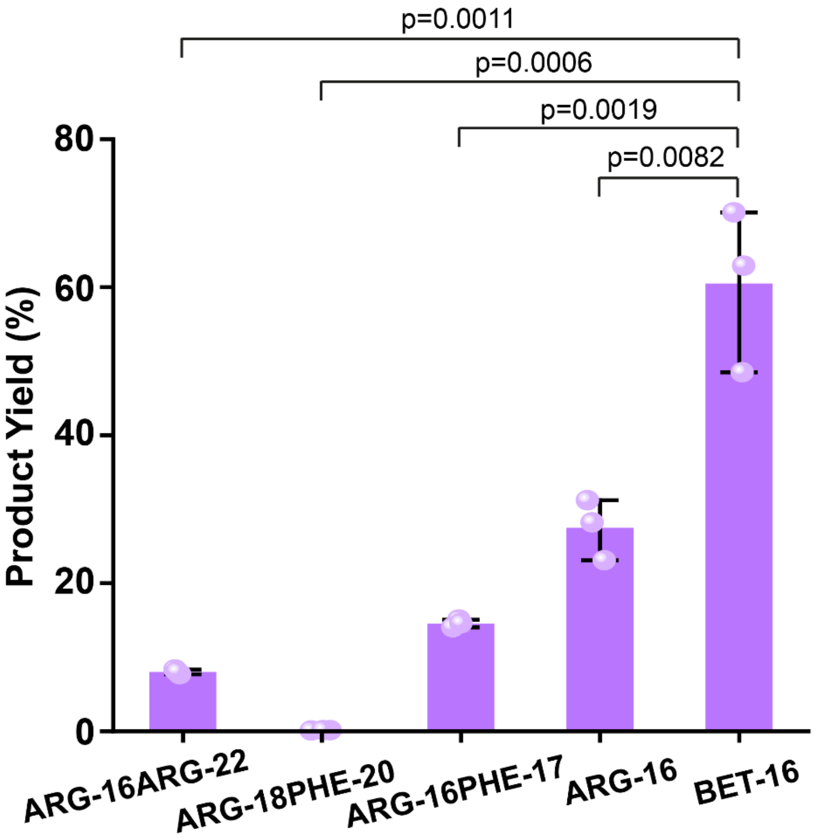

Supplementary Figure 15. Comparison of product (2) yield by different peptide sequences. Bar diagram showing the yield of alcohol (**2**) by different peptide sequences in presence of $NaBH_4$ in 12 h. The error bars are calculated from three separate experiments. Data are presented as the mean ± s.d. (n=3 independent experiments). Significance was estimated using a two-sided homoscedastic ‘t’ test (*p ≤ 0.05, **p ≤ 0.01, ***p ≤ 0.001). Source data are provided as a Source Data file.

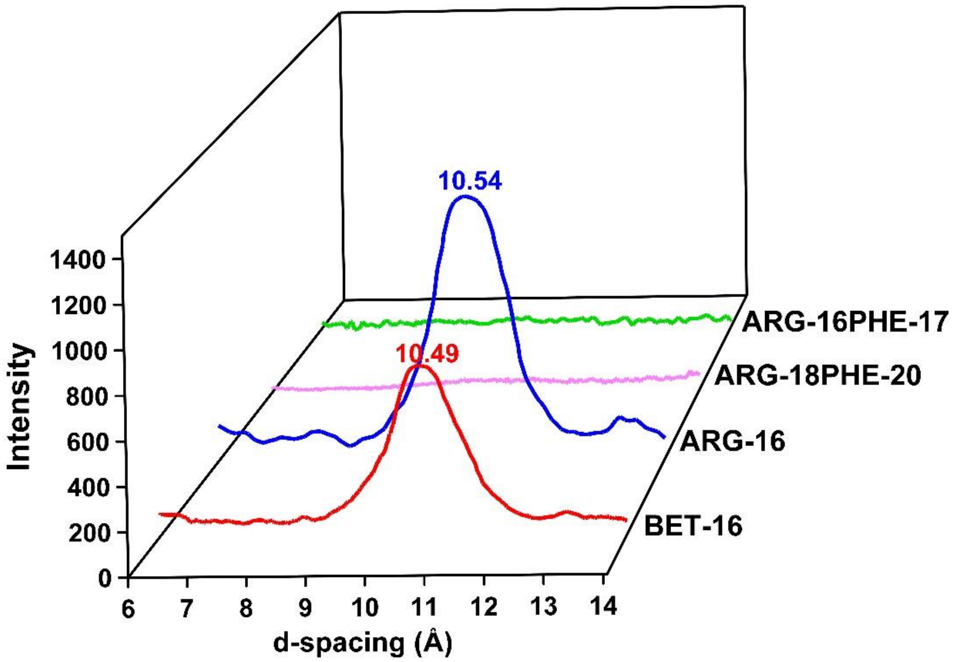

Supplementary Figure 16. **PXRD profiles for different peptide assemblies.** Comparison of the PXRD profiles for the β-sheet laminate distances of BET-16, ARG-16, ARG-16PHE-17 and ARG-18PHE-20. Source data are provided as a Source Data file.

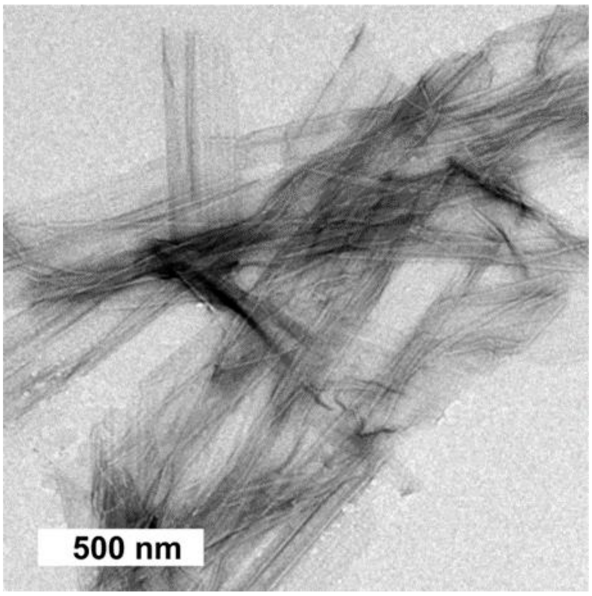

Supplementary Figure 17. **Characterization of the morphology of GLU-16 assemblies.** TEM micrograph of GLU-16. All the experiments were repeated for at least three times.

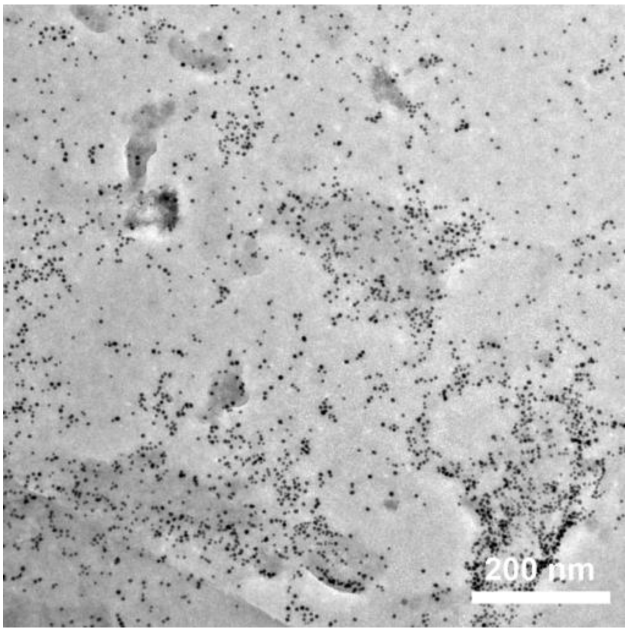

Supplementary Figure 18. **Characterization of GNP_{neg} bound GLU-16 assemblies**. TEM micrograph of GNP_{neg} in presence of GLU-16. All the experiments were repeated for at least three times.

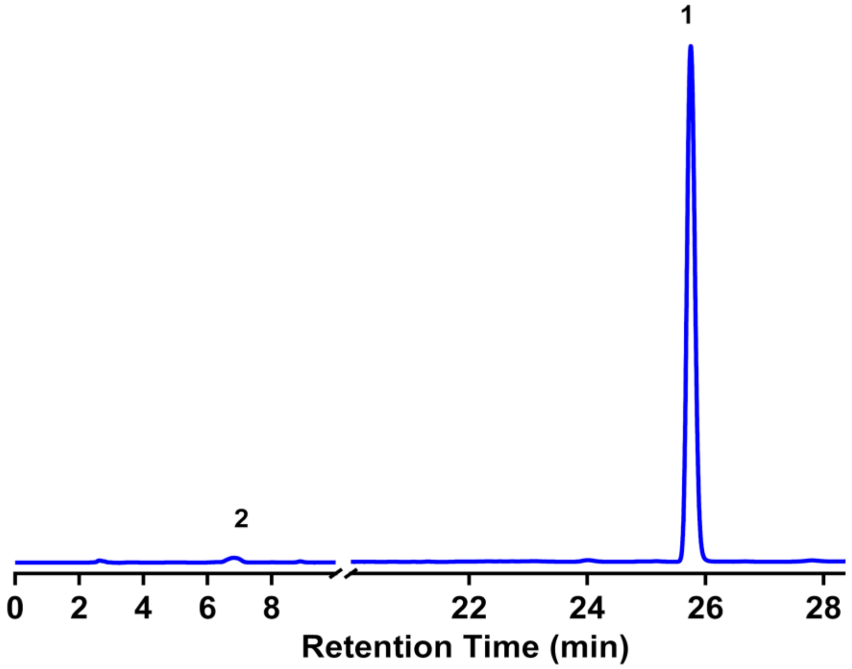

Supplementary Figure 19. **Characterization of the reduction of 1 by GLU-16-$NaBH_4$ system from HPLC analysis**. HPLC chromatogram of the reduction of **1** by GLU-16-$NaBH_4$ system at 12 h (pH 10), extracted at 276 nm. Source data are provided as a Source Data file.

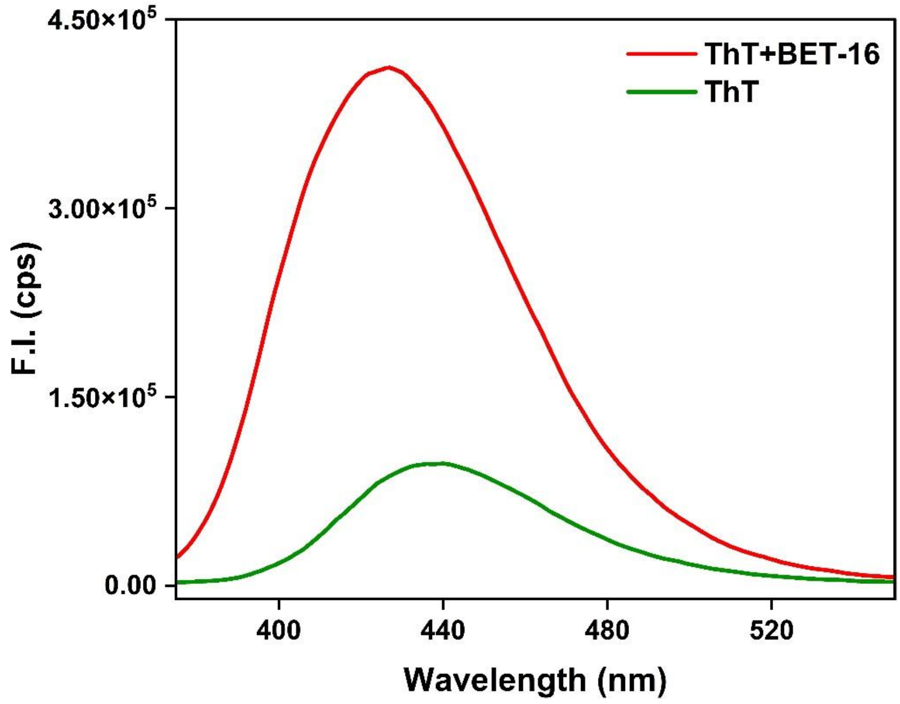

Supplementary Figure 20. **ThT binding assay of BET-16 assemblies.** Fluorescence emission spectra of ThT in presence or absence of BET-16. Source data are provided as a Source Data file.

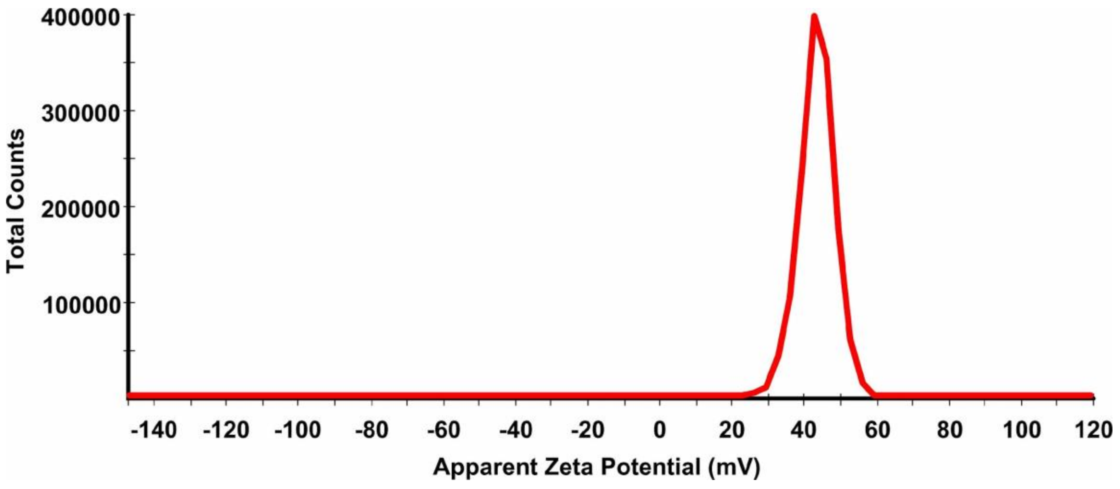

Supplementary Figure 21. **Characterization of the cationic BET-16 assemblies**. Zeta potential of BET-16 assemblies.

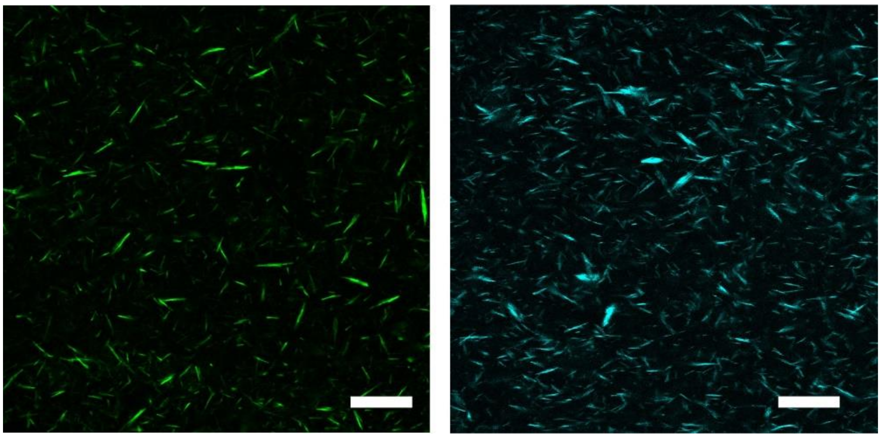

Supplementary Figure 22. **Characterization of BET-16 assemblies in presence of different dyes**. CLSM images of BET-16 in presence of FITC (left) and RITC (right). Scale bar = 20 μm. All the experiments were repeated for at least three times.

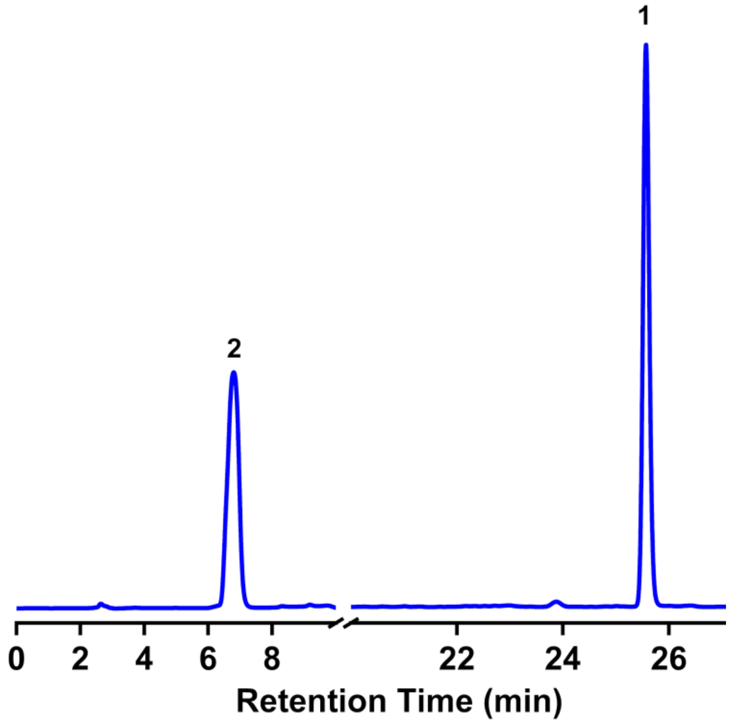

Supplementary Figure 23. Characterization of the reduction of 1 by BET-16-NaBH$_4$ system from HPLC analysis. HPLC chromatogram of the reduction of **1** by BET-16-NaBH$_4$ system at 12 h (pH 10), extracted at 276 nm. Source data are provided as a Source Data file.

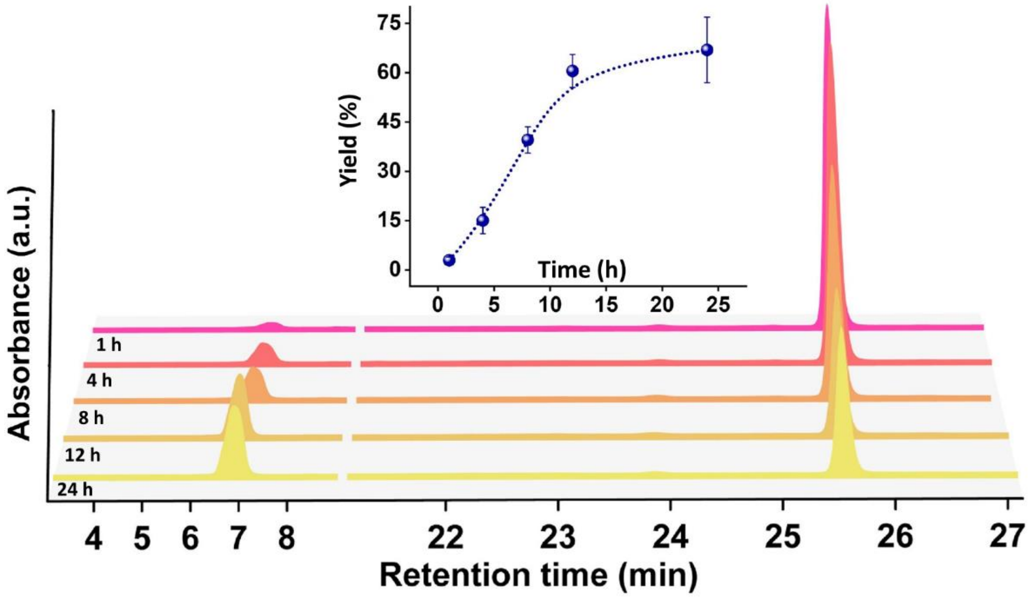

Supplementary Figure 24. Characterization of the time-dependent generation of product (2) of the reduction of 1 by BET-16-NaBH$_4$ system. Time resolved HPLC chromatograms of the reduction reaction of **1** and the percentage of product (**2**) yield by BET-16. The error bars are calculated from three separate experiments. Data are presented as the mean ± s.d. (n=3 independent experiments). Source data are provided as a Source Data file.

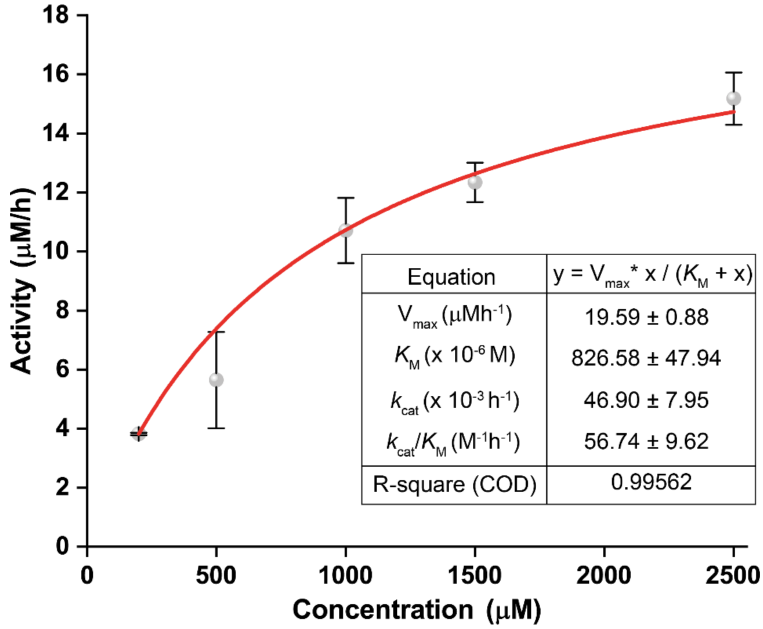

Supplementary Figure 25. **Detailed kinetic analysis of the reduction of 1 by BET-16-NaBH$_4$ system**. Representative Michaelis-Menten plot for BET-16 (500 μM) with varying concentrations of **1**, [NaBH$_4$] =10 mM. Error bars are calculated from three separate experiments. Data are presented as the mean ± s.d. (n=3 independent experiments). Source data are provided as a Source Data file.

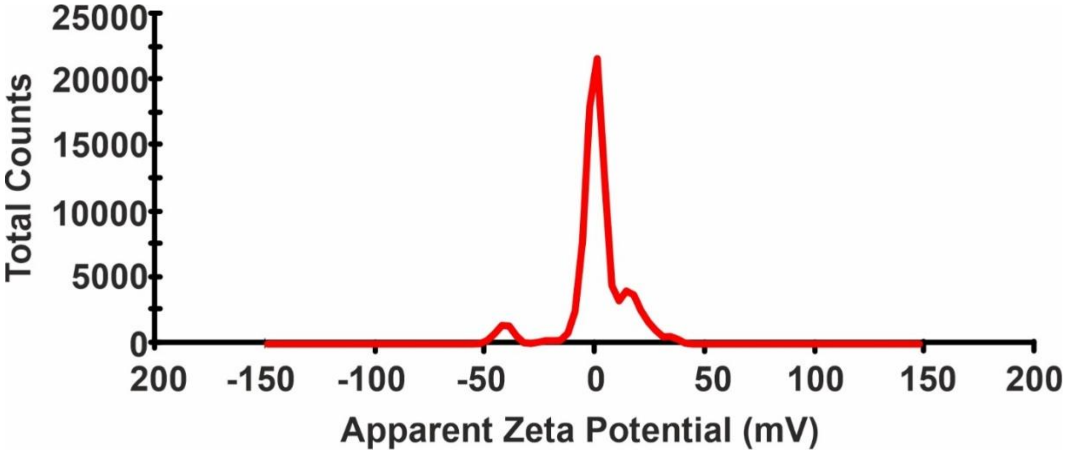

Supplementary Figure 26. Characterization of ARG-16 assemblies in presence of higher concentration of cofactor $NaBH_4$. Zeta potential of ARG-16 in presence of 30 mM $NaBH_4$.

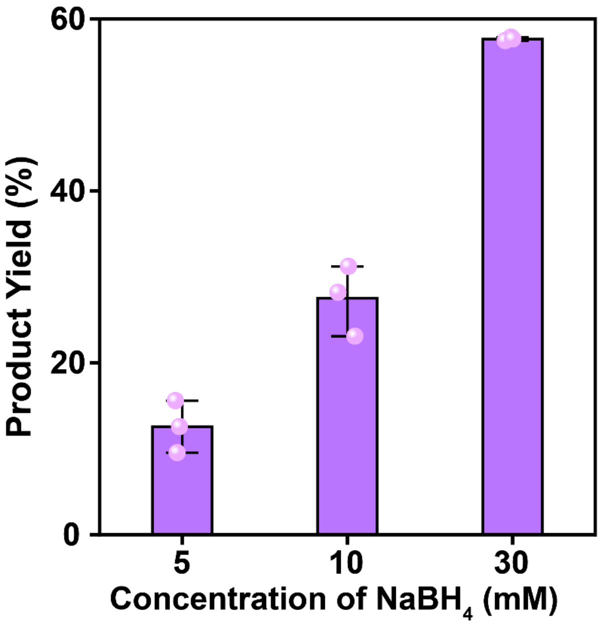

Supplementary Figure 27. The effect of cofactor concentration on the product yield of reduction of 1 by ARG-16- $NaBH_4$ system. Bar diagram showing the product (**2**) yield by ARG-16 at 12 h with increasing concentrations of $NaBH_4$. The error bars are calculated from three separate experiments. Data are presented as the mean ± s.d. (n=3 independent experiments). Source data are provided as a Source Data file.

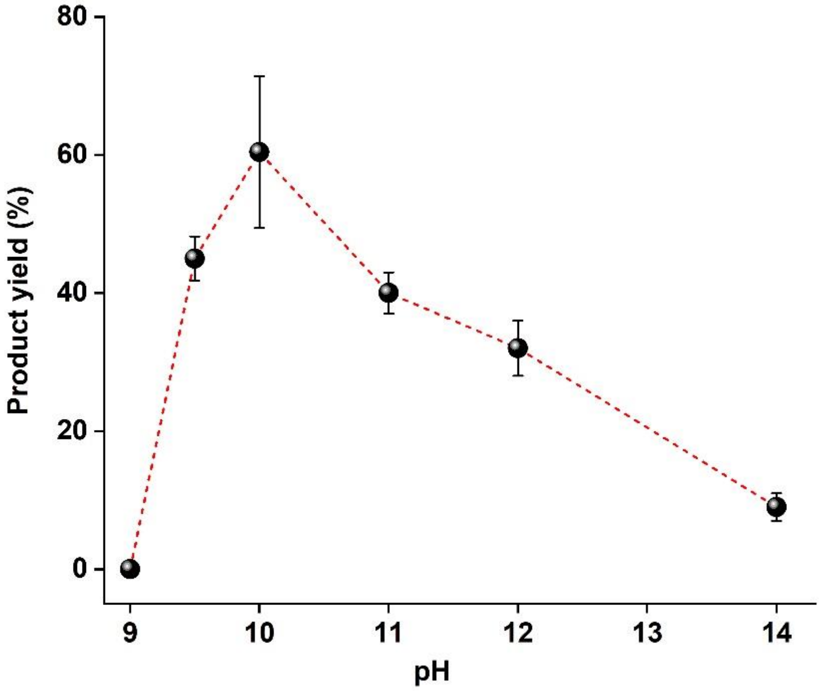

Supplementary Figure 28. **The effect of pH on the reduction reaction of 1 by BET-16-$NaBH_4$ system.** The effect of pH on the yield of **2** by cofactor bound BET-16 assemblies. NaOH/HCl was added to adjust the respective pH value of the reaction mixture. The error bars are calculated from three separate experiments. Data are presented as the mean ± s.d. (n=3 independent experiments). Source data are provided as a Source Data file.

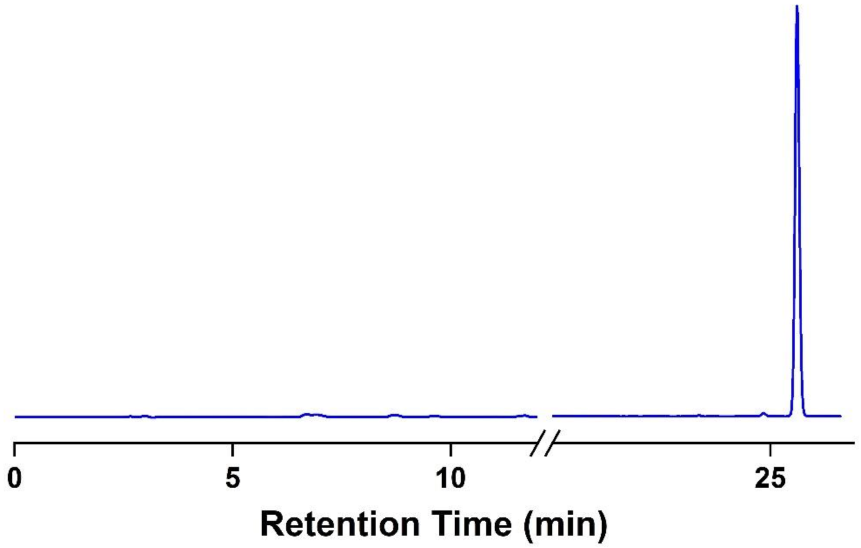

Supplementary Figure 29. **Characterization of reduction reaction of 1 by BET-16- $NaBH_4$ system at pH 7.** HPLC chromatogram of the reduction of **1** by BET-16-$NaBH_4$ system at pH 7 after 12 h, extracted at 276 nm. Source data are provided as a Source Data file.

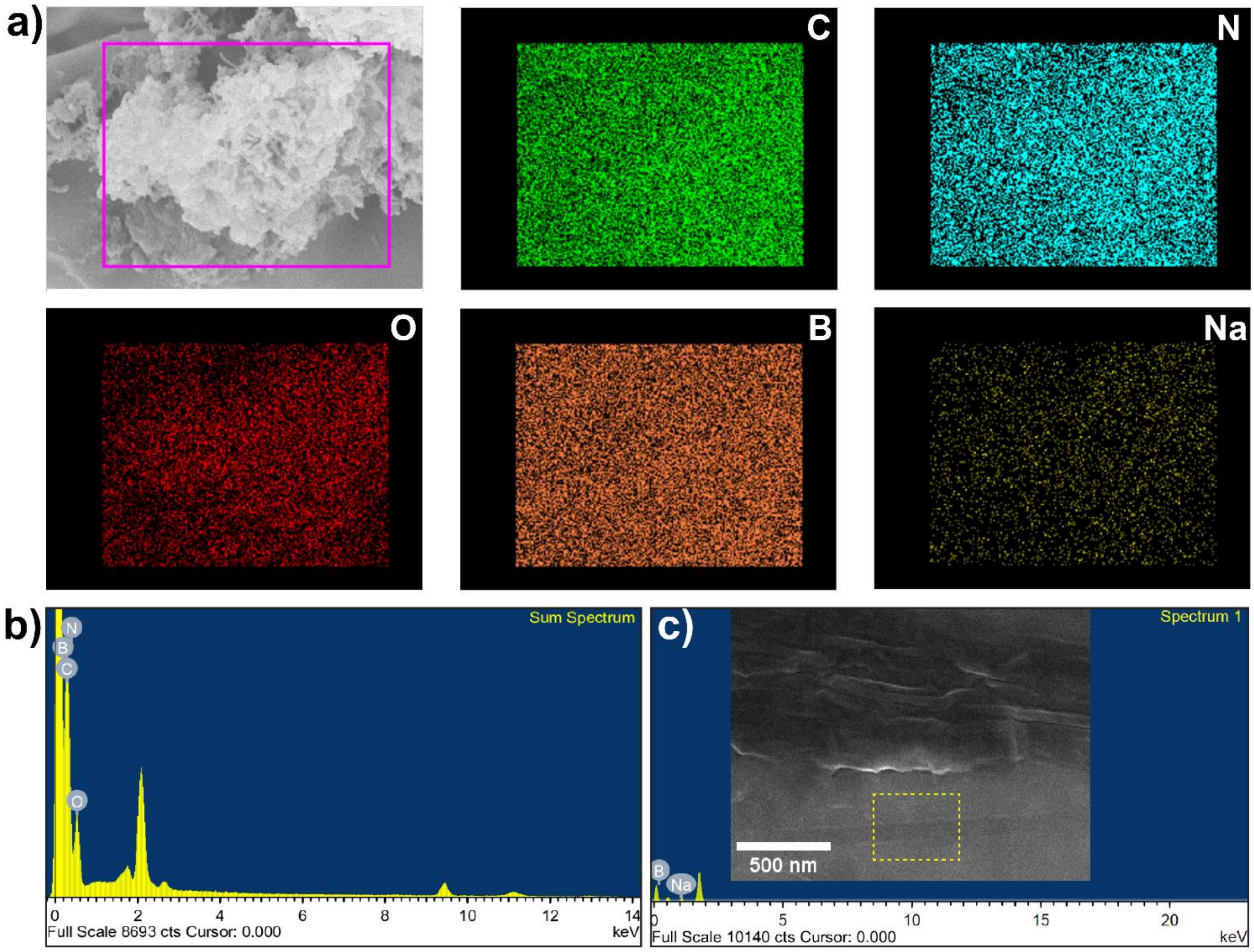

Supplementary Figure 30. **SEM-EDS analysis of cofactor-bound BET-16 assemblies.** a) SEM cross-sectional image of the lyophilized powder of cofactor-bound BET-16 and the elemental mapping of C, N, O, B and Na. b) EDS spectrum of the lyophilized powder of cofactor-bound BET-16 assemblies. c) EDS spectrum showing the elemental mapping of B and Na on the cofactor-bound BET-16 nanotubes (excluding the highly intense peaks of the elements C, O, N and Si). Inset shows the corresponding SEM image and the elemental mapping region (yellow dotted line) of the BET-16 assemblies.

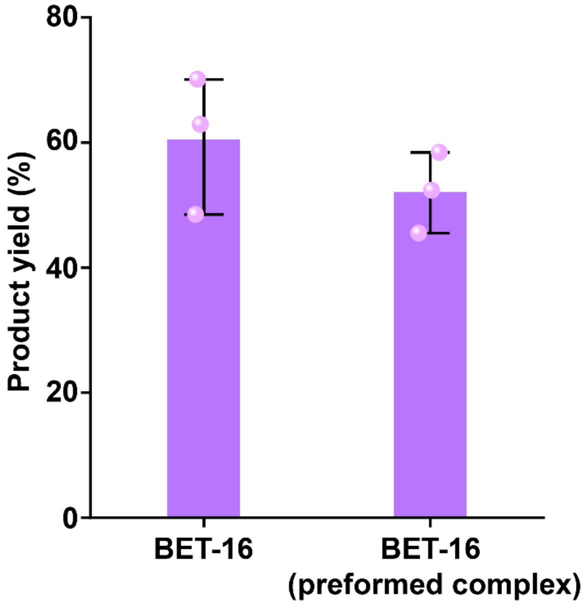

Supplementary Figure 31. **The effect of pre-complexation of cofactor with BET-16 assemblies on the product yield of reduction of 1.** Bar diagram showing the product yield for the preformed complex of cofactor ($NaBH_4$) bound BET-16 assemblies compared to the main system. The error bars are calculated from three separate experiments. Data are presented as the mean ± s.d. (n=3 independent experiments). Source data are provided as a Source Data file.

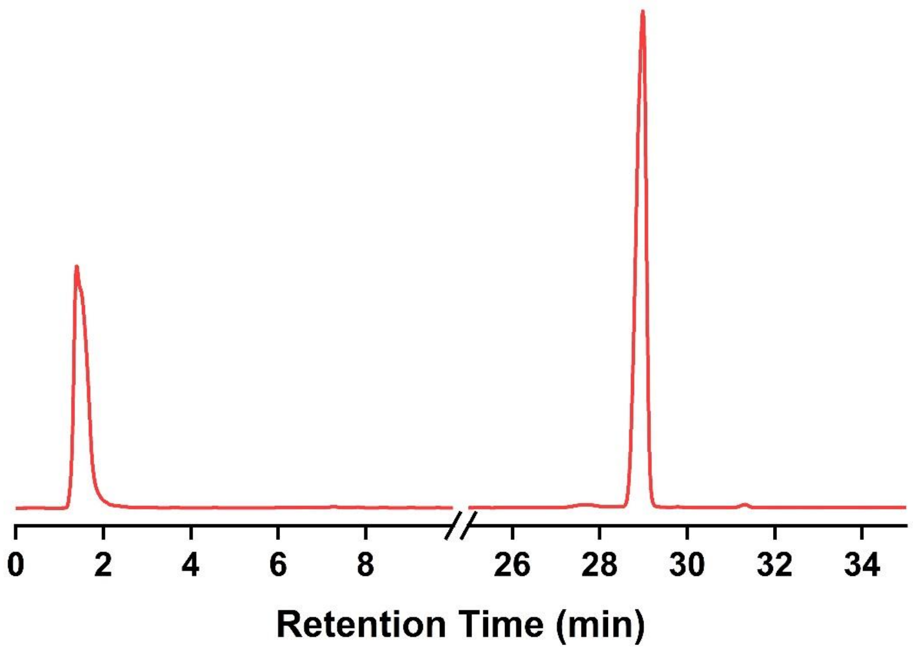

Supplementary Figure 32. **Characterization of the reduction of 1 by BET-16 assemblies in presence of NADH.** HPLC chromatogram of the reduction of **1** by BET-16-NADH system at 12 h (pH 10), extracted at 276 nm. The peak at 1.4 min corresponds to NADH and the peak at 29 min corresponds to the unreacted ester **1**. Source data are provided as a Source Data file.

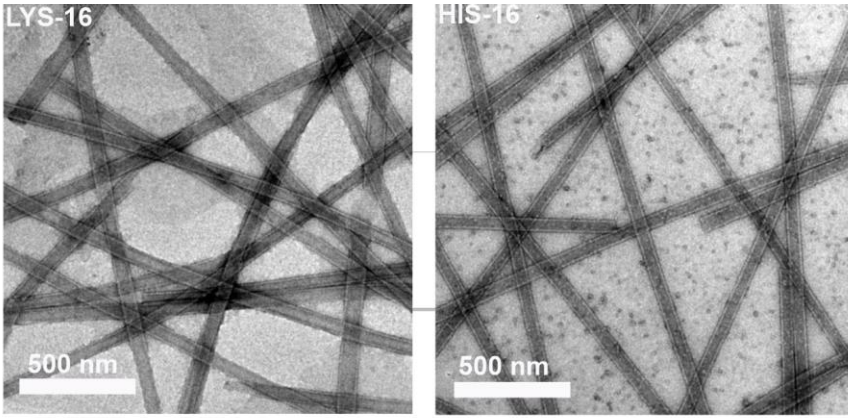

Supplementary Figure 33. Detection of the morphologies of different assemblies. TEM micrographs of LYS-16 (left) and HIS-16 (right). All the experiments were repeated for at least three times.

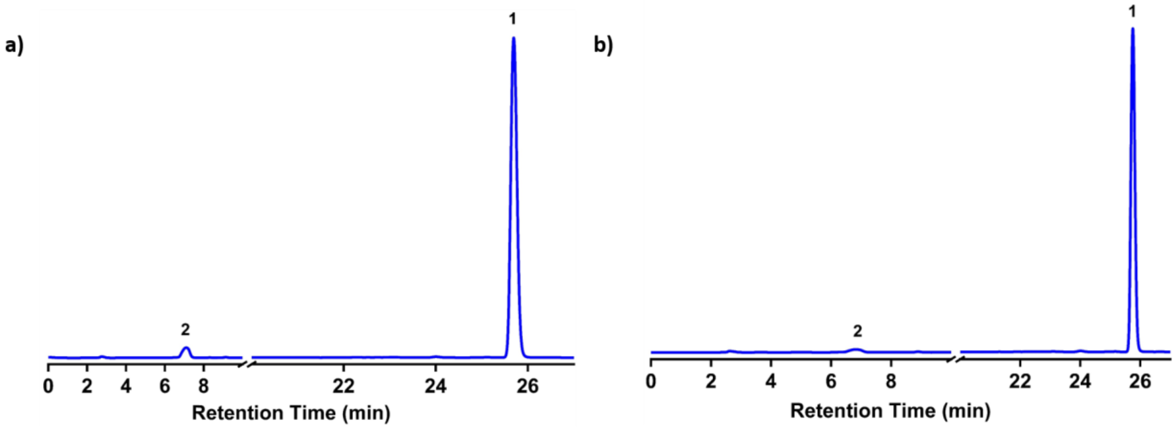

Supplementary Figure 34. **Characterization of reduction of 1 by different assemblies.** HPLC chromatograms of the reduction of **1** by a) LYS-16-$NaBH_4$ system and b) HIS-16-$NaBH_4$ system (pH 10) at 12 h, extracted at 276 nm. Source data are provided as a Source Data file.

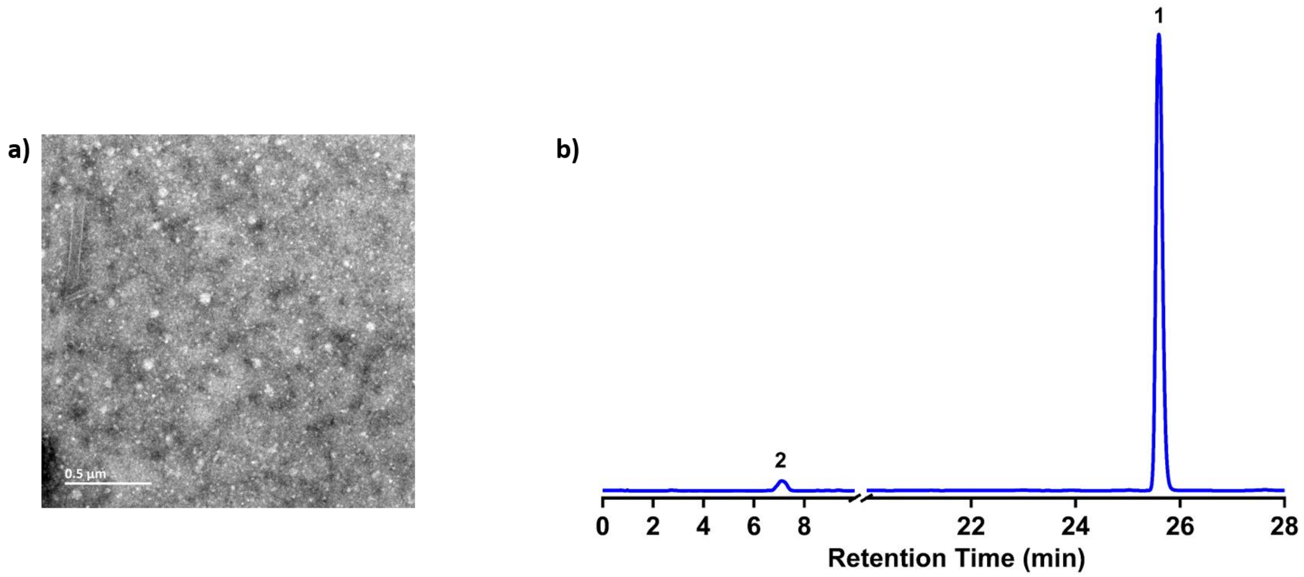

Supplementary Figure 35. Characterization of HFIP-treated disassembled BET-16 assemblies and their effect on reduction of 1. a) TEM micrograph of BET-16 treated with HFIP. The experiment was repeated for at least three times. b) HPLC chromatogram of reduction reaction mixture containing BET-16-HFIP-$NaBH_4$ system at 12 h, extracted at 276 nm**.** Source data are provided as a Source Data file.

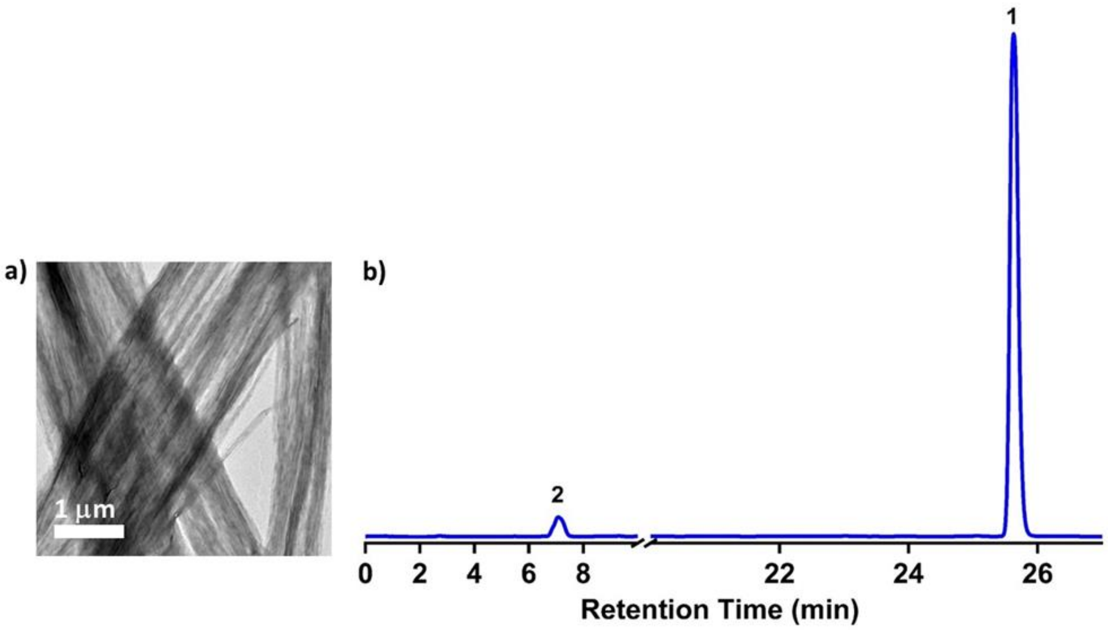

Supplementary Figure 36. Characterization of Na_2SO_4-treated bundled BET-16 assemblies and their effect on reduction of 1. a) TEM micrograph of BET-16 with Na_2SO_4. The experiment was repeated for at least three times. b) HPLC chromatogram of the reduction reaction by BET-16-$NaBH_4$-Na_2SO_4 system at 12 h, extracted at 276 nm**.** Source data are provided as a Source Data file.

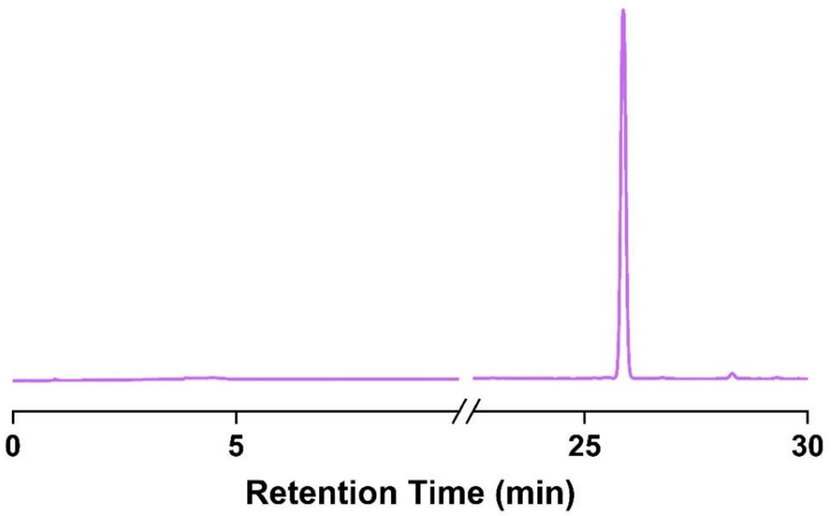

Supplementary Figure 37. Effect of cofactor on reduction of 1. HPLC chromatogram of the reaction mixture of **1** and BET-16 at 12 h in absence of $NaBH_4$ (pH 10 was maintained by adding few microlitres of NaOH). Source data are provided as a Source Data file.

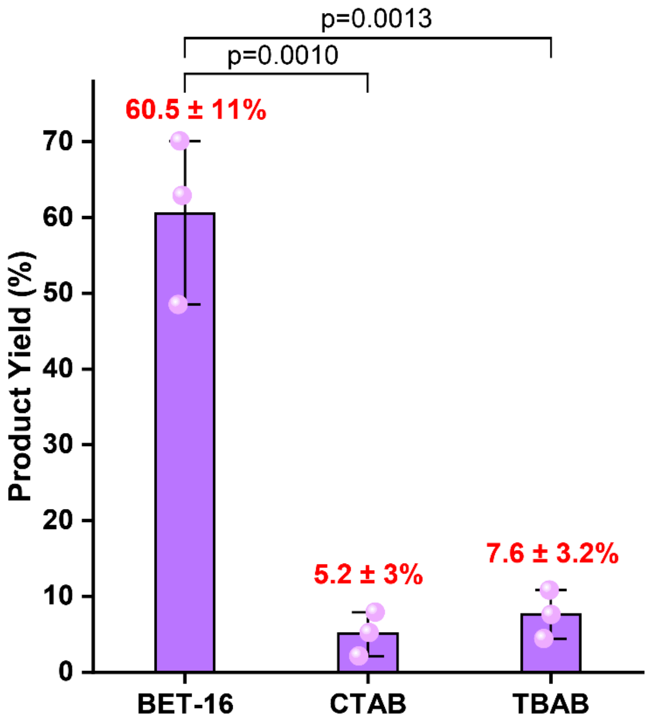

Supplementary Figure 38. Comparison of product conversions by different systems. Bar diagram showing the yield of **2** by BET-16-$NaBH_4$ system, CTAB in presence of $NaBH_4$ and by tetrabutylammonium borohydride (TBAB) system in 12 h. The error bars are calculated from three separate experiments. Data are presented as the mean ± s.d. (n=3 independent experiments). Significance was estimated using a two-sided homoscedastic 't' test (*$p \leq 0.05$, **$p \leq 0.01$, ***$p \leq 0.001$). Source data are provided as a Source Data file.

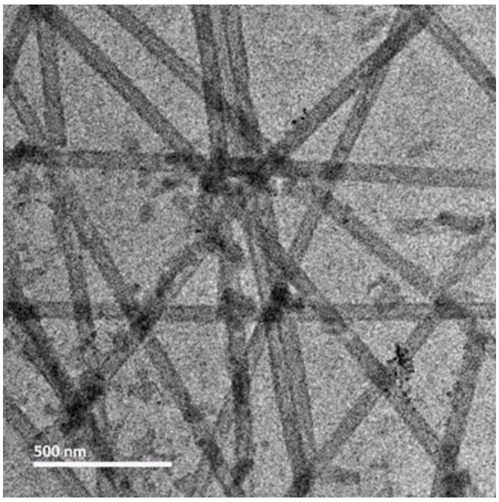

Supplementary Figure 39. Retention of BET-16 nanotubular morphologies after reaction cycles. Retention of nanotubular morphologies of BET-16 after the completion of two cycles of reaction (pH of the reaction mixture was 10). The experiment was repeated for at least three times.

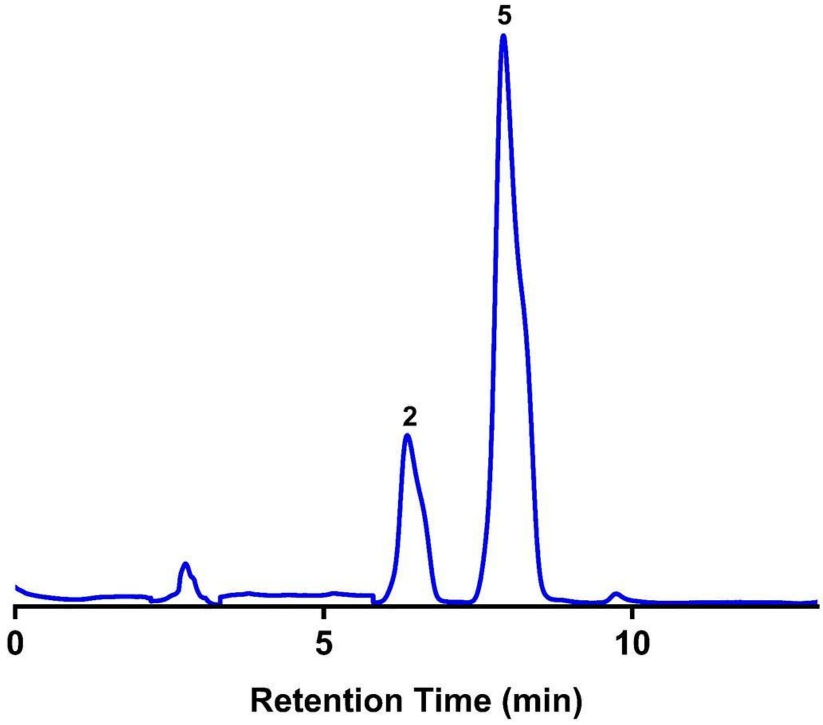

Supplementary Figure 40. Characterization of reduction of 5. HPLC chromatogram of reduction of **5** by BET-16-$NaBH_4$ system at 12 h, extracted at 276 nm. Source data are provided as a Source Data file.

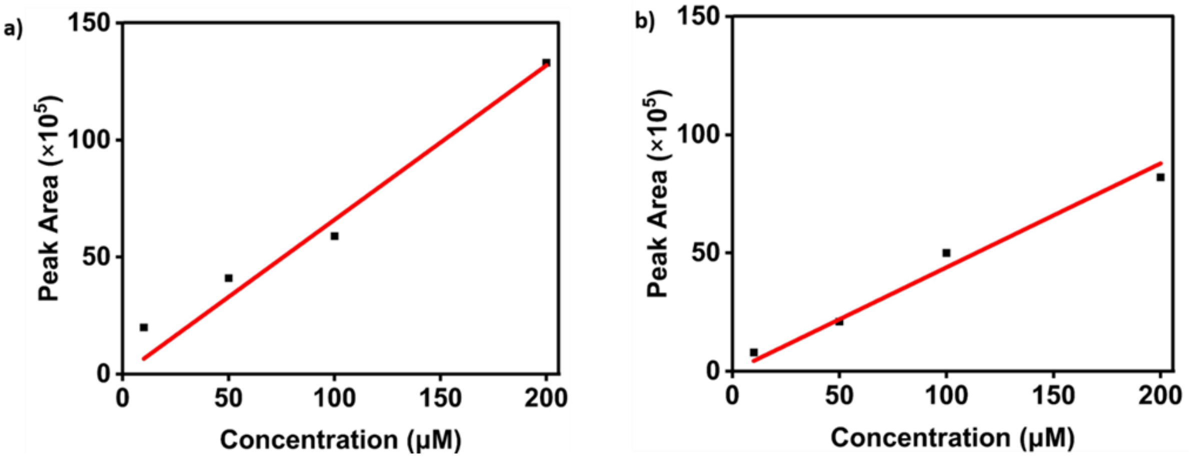

Supplementary Figure 41. Calibration curves of 1 and 2. a) Standard curve of peak area vs concentration of **1.** b) Standard curve of peak area vs concentration of **2**, extracted at 276 nm. Source data are provided as a Source Data file.

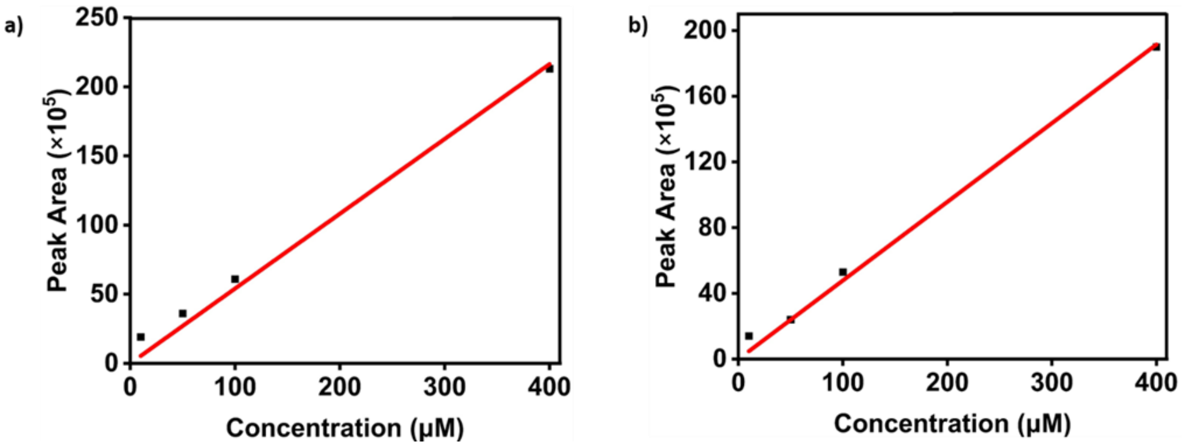

Supplementary Figure 42. Calibration curves of 3 and 4. a) Standard curve of peak area versus concentration of **3.** b) Standard curve of peak area versus concentration of **4**, extracted at 276 nm. Source data are provided as a Source Data file.

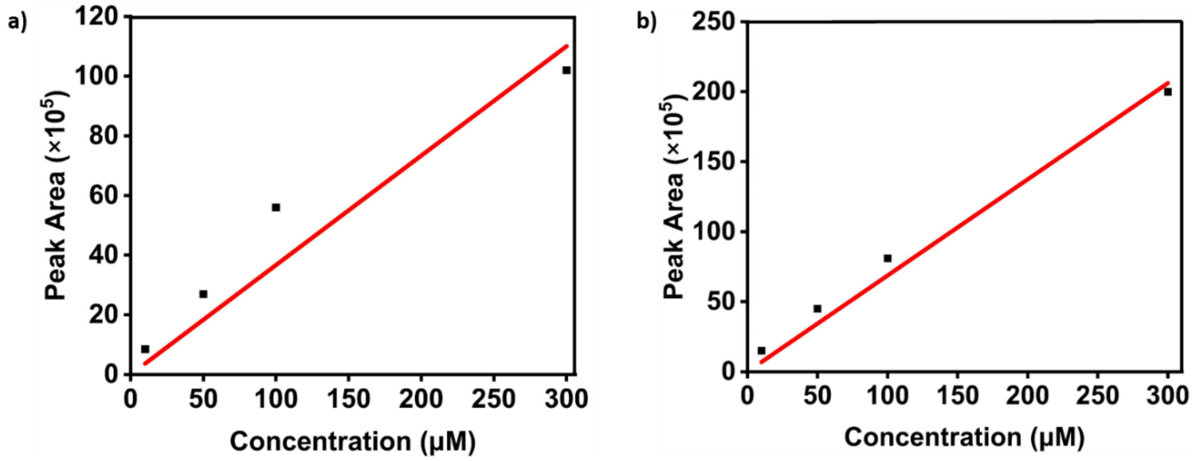

Supplementary Figure 43. Calibration curves of 5 and 6. a) Standard curve of peak area versus concentration of **5.** b) Standard curve of peak area versus concentration of **6**, extracted at 276 nm. Source data are provided as a Source Data file.

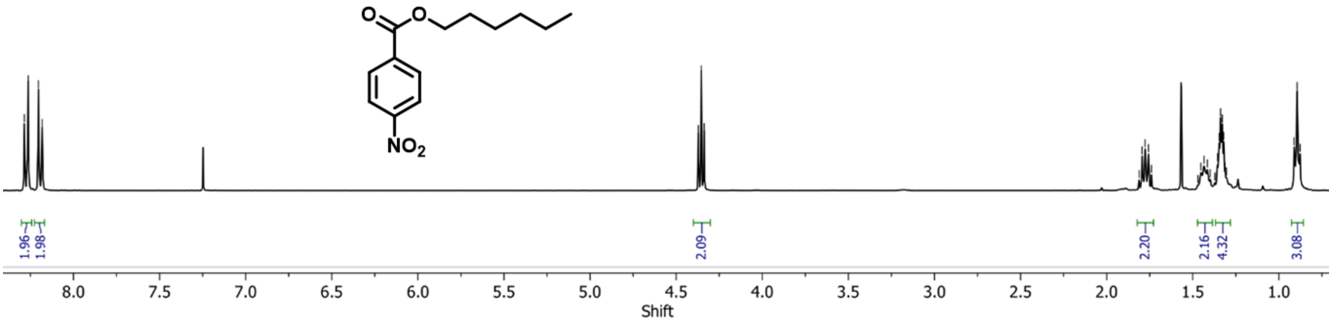

Supplementary Figure 44. ^{1}H NMR spectrum of **1**.

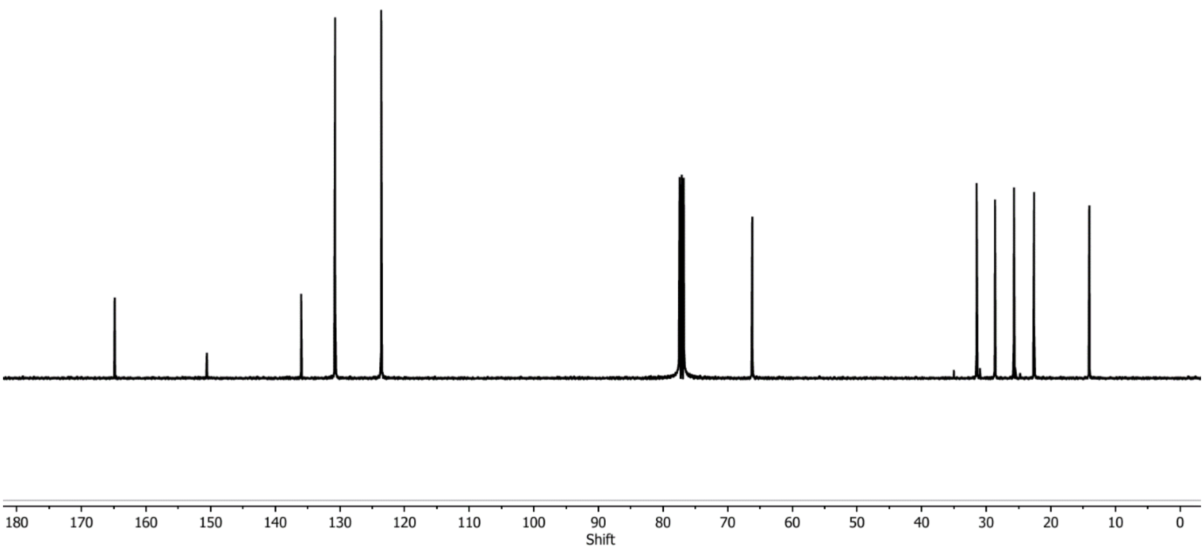

Supplementary Figure 45. ^{13}C NMR spectrum of **1**.

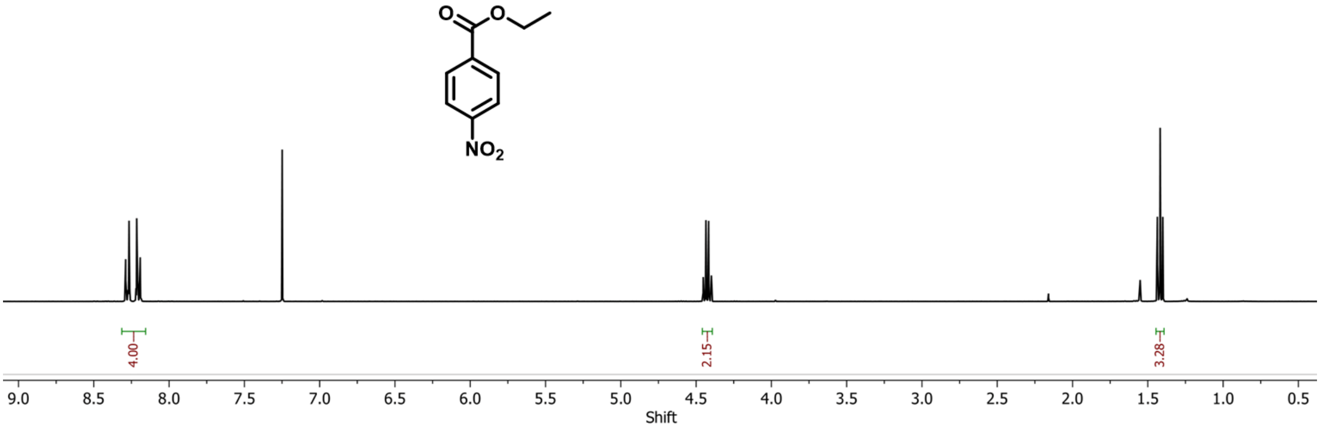

Supplementary Figure 46. ^{1}H NMR spectrum of **3**.

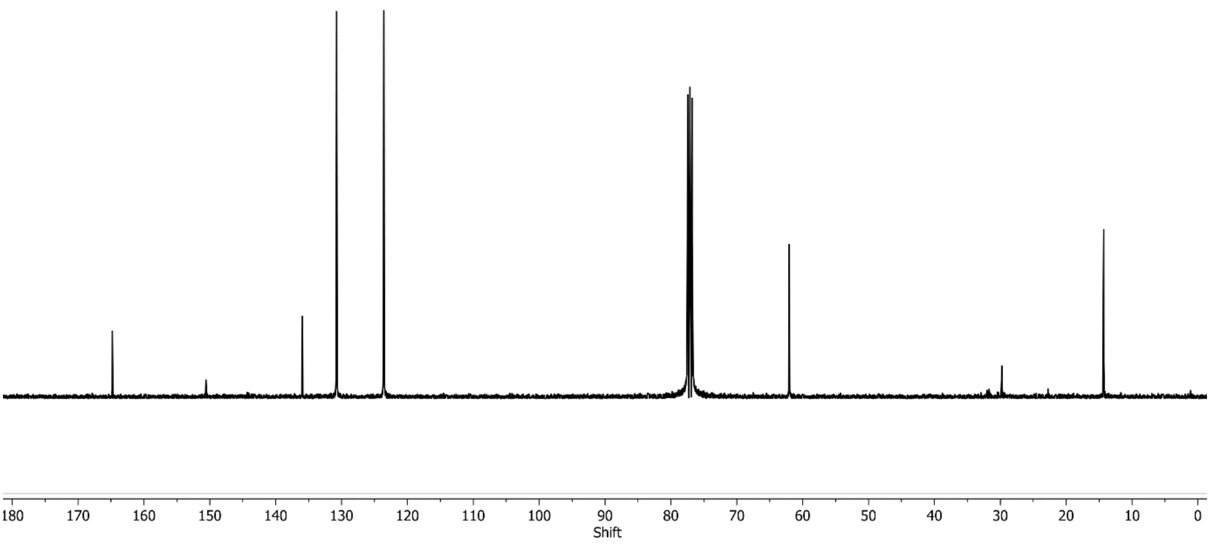

Supplementary Figure 47. ^{13}C NMR spectrum of **3**.

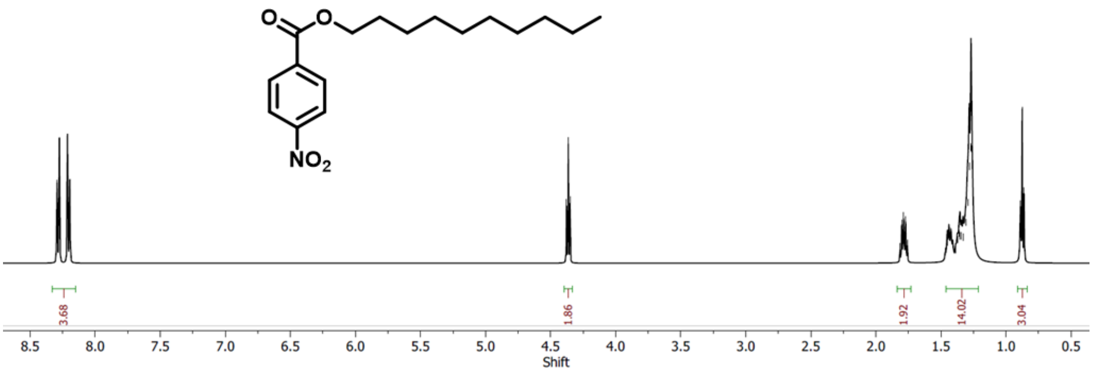

Supplementary Figure 48. ^{1}H NMR spectrum of **4**.

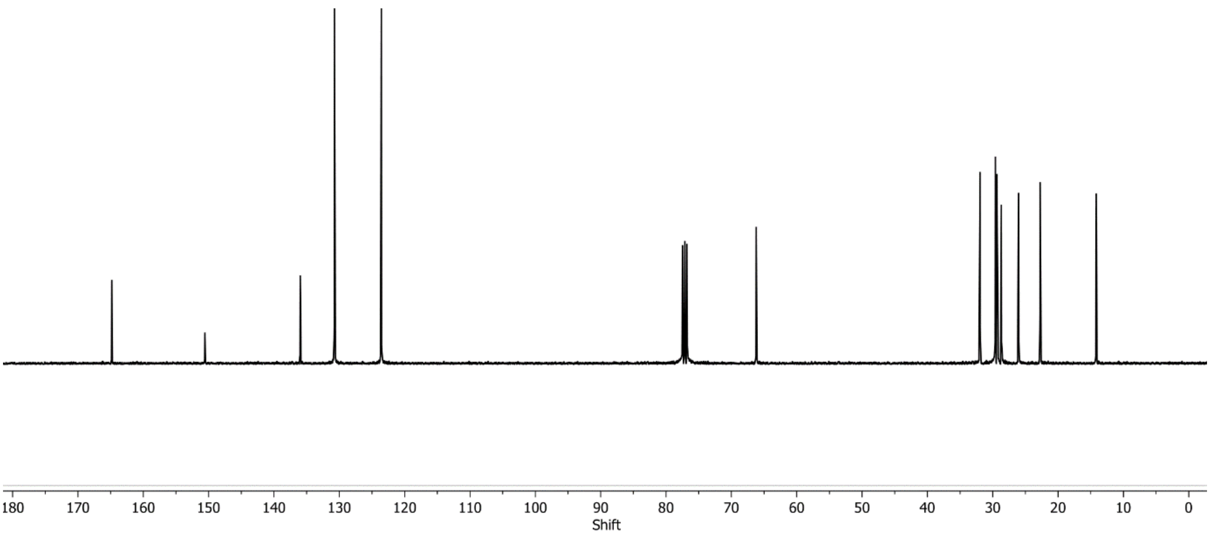

Supplementary Figure 49. ^{13}C NMR spectrum of **4**.

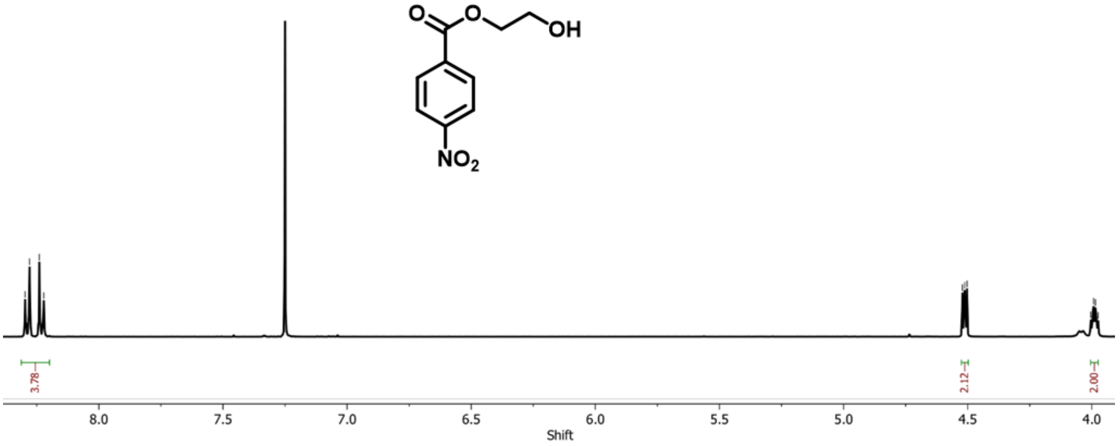

Supplementary Figure 50. ^{1}H NMR spectrum of **5**.

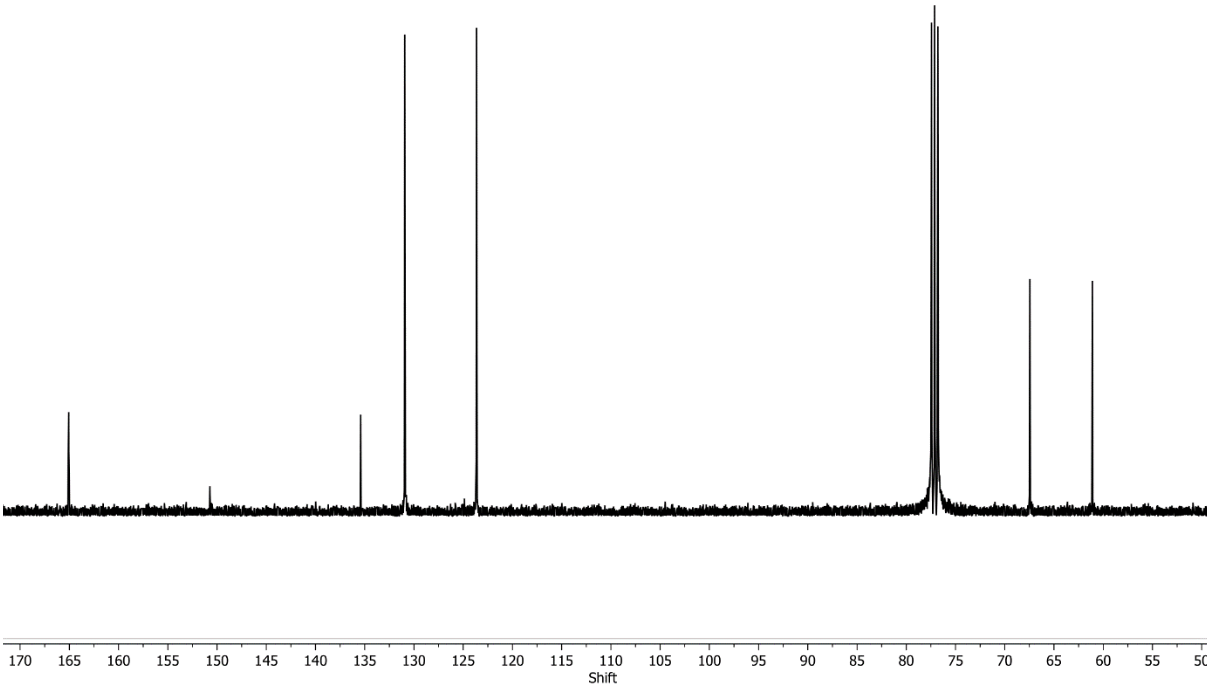

Supplementary Figure 51. ^{13}C NMR spectrum of **5**.

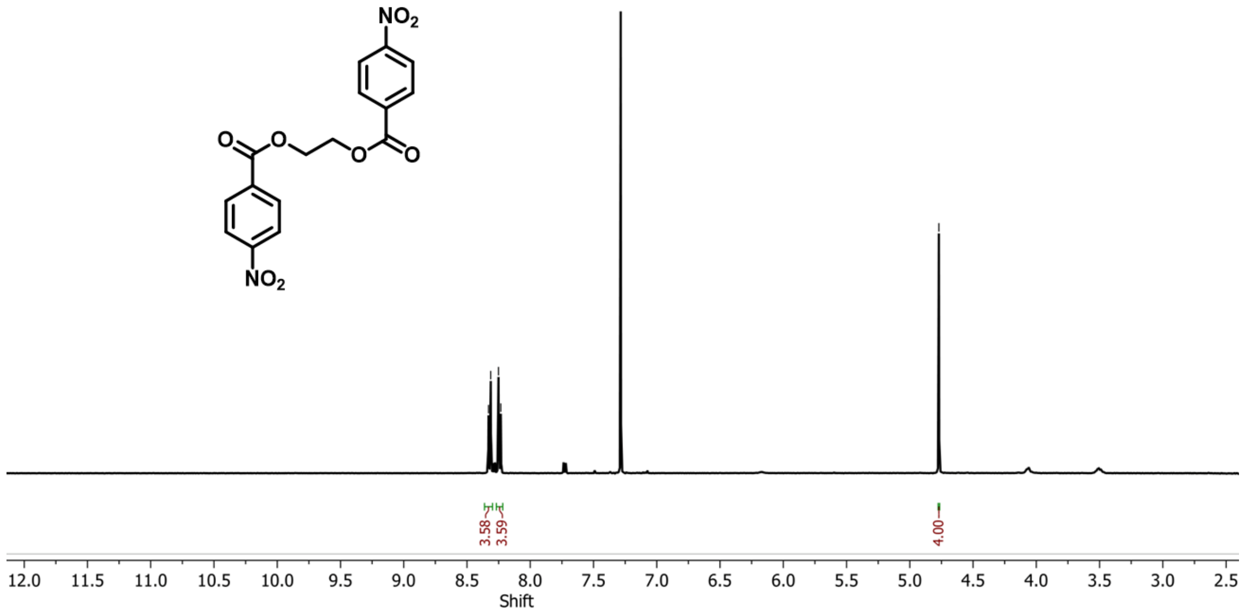

Supplementary Figure 52. ^{1}H NMR spectrum of **6**.

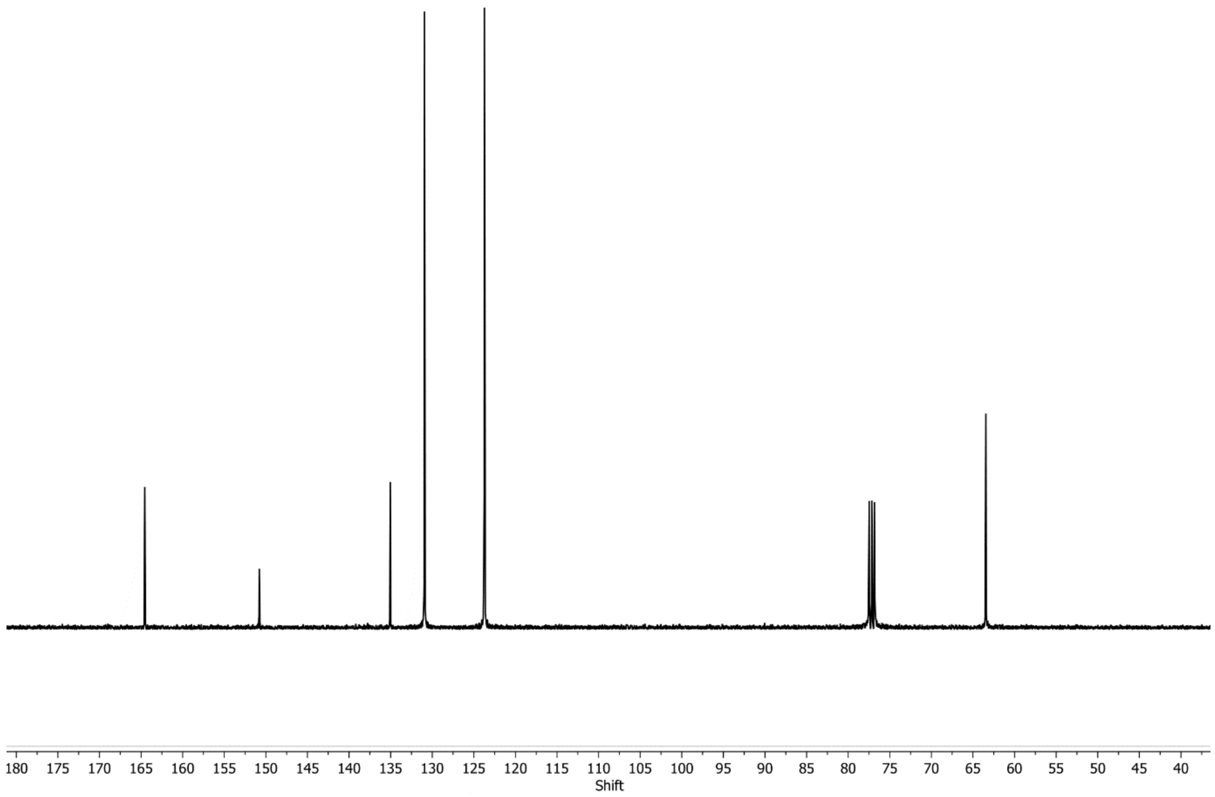

Supplementary Figure 53. ^{13}C NMR spectrum of **6**.

Supplementary References

1. B. Sarkhel, A. Chatterjee, D. Das. Covalent Catalysis by Cross β Amyloid Nanotubes. *J. Am. Chem. Soc.* **142**, 4098-4103 (2020).
2. B. Neises, W. Steglich. Simple Method for the Esterification of Carboxylic Acids. *Angew. Chem.* **90,** 556-557 (1978).
3. Schlesinger, H. I. et al. Sodium Borohydride, Its Hydrolysis and its Use as a Reducing Agent and in the Generation of Hydrogen. *J. Am. Chem. Soc.* **75**, 215-219 (1953).
